# Engineering *Gypsophila elegans* hairy root cultures to produce endosomal escape‐enhancing saponins

**DOI:** 10.1111/pbi.70122

**Published:** 2025-05-10

**Authors:** Elia Lacchini, Tongtong Qu, Tessa Moses, Alexander N. Volkov, Alain Goossens

**Affiliations:** ^1^ Department of Plant Biotechnology and Bioinformatics Ghent University Ghent Belgium; ^2^ VIB Center for Plant Systems Biology Ghent Belgium; ^3^ Department of Plant Molecular Biology University of Lausanne Lausanne Switzerland; ^4^ EdinOmics, RRID:SCR_021838 University of Edinburgh Edinburgh UK; ^5^ VIB‐VUB Center for Structural Biology Brussels Belgium; ^6^ Jean Jeener NMR Centre, VUB Brussels Belgium; ^7^ Department of Botany and Zoology Stellenbosch University Matieland South Africa

**Keywords:** triterpenoid saponins, CYP450, hairy roots, endosomal escape, oleanane, β‐amyrin

## Abstract

The limited cytosolic delivery of DNA and protein‐based therapeutics due to endosomal entrapment reduces drug efficacy, increasing treatment costs and possible side effects in human and veterinary medicine as a consequence of higher administered dosages. Plant‐derived triterpenoid saponins, specifically those with endosomal escape‐enhancing (EEE) properties, have shown promise in overcoming this limitation by disrupting endosomal membranes. QS‐21, a well‐known EEE saponin, has been used as an adjuvant in vaccines, and recent studies have elucidated its biosynthetic pathway. However, EEE saponins are typically present as minor compounds in plants, posing challenges for their large‐scale production and purification. Here we investigated the possibility of engineering SO1861 production, an EEE saponin from *Saponaria officinalis*, using heterologous gene expression in *Gypsophila elegans* hairy roots, a plant species known to synthesize structurally related saponins. Via *S. officinalis* transcriptomics, we identified jasmonate‐responsive saponin biosynthetic genes, and three cytochrome P450s (CYP450s) involved in C23, C28 and C16 oxidations were characterised. Heterologous expression of these CYP450s in *G. elegans* hairy roots successfully altered the saponin profile, with notable increases in SO1861 precursors in lines expressing the C23‐oxidases SoCYP72A984 and SoCYP72A1003. Interestingly, expression of only SoCYP72A1003, a non‐canonical C23 oxidase, resulted in the accumulation of a compound matching the SO1861 standard, suggesting the activation of a potentially latent pathway and of silent enzymes in a novel combination. This work underscores the potential of engineering strategies in heterologous plant systems to steer triterpenoid saponin biosynthetic pathways and suggests new avenues for producing high‐value EEE saponins.

## Introduction

The adoption of potent DNA/protein‐based drugs in modern targeted therapeutics is frequently hampered by scarce cytosolic delivery (Ebrahimi and Samanta, 2023; Xie *et al*., 2023). This limitation arises from the endocytic uptake of these drugs, which results in their entrapment within endosomes (Voltà‐Durán *et al*., 2023). These vesicles typically follow one of two fates: they are either recycled back to the cell surface and secreted, or they fuse with lysosomes, leading either to secretion or degradation of their contents (Voltà‐Durán *et al*., 2023). In both cases, only a small fraction of the administered drug reaches the cytosol to exert its function (Dowdy, 2023). This has an impact on efficacy, cost of treatment, and potential side effects due to increased drug dosage.

Recent research has identified highly glycosylated triterpenoid saponins derived from plants as a promising solution to the challenge posed by endosomal trapping (Fuchs *et al*., 2017; Sama *et al*., 2017, 2018). When co‐administered with the drug, these saponins can destabilize endosomal membranes, causing their rupture and thus the leakage of the endosomal content into the cytosol (Fuchs *et al*., 2017). This process, known as endosomal escape (Voltà‐Durán *et al*., 2023), can therefore greatly enhance treatment efficacy (Sama *et al*., 2017), and the saponins able to induce this process are referred to as endosomal escape‐enhancing (EEE) saponins or endosomal escape enhancers.

The structure–function relationships common to triterpenoid saponins with EEE properties have been elucidated. They reveal a hydrophobic oleanane‐derived pentacyclic backbone featuring an aldehyde group at C‐23, a hydrophilic oligosaccharide ester chain bound at C‐28, and a branched sugar chain at C‐3, beginning with a glucuronic acid moiety (Bachran *et al*., 2006). These key features characterise one of the most notable examples of an EEE saponin: QS‐21, a highly glycosylated triterpenoid saponin extracted from the bark of the *Quillaja saponaria* tree, native to South America (Martin *et al*., 2024). QS‐21 has gained significant attention in recent years due to its ability to enhance both antibody‐ and cell‐mediated immune responses. The importance of QS‐21 in modern medicine is underscored by its incorporation into various vaccines, including those against malaria (Mosquirix™), Herpes zoster (Shingrix™), and COVID‐19 (NVX‐CoV2373™ Novavax). This wide application demonstrates the potential of EEE saponins to improve the efficacy of not only targeted therapeutics, but also vaccine formulations.

Besides Quillaja, several plant species (Koczurkiewicz‐Adamczyk *et al*., 2023; Sama *et al*., 2018), especially members of the *Caryophyllaceae*, are reported to have an extensive repertoire of molecules with endosomal escape properties (Jo *et al*., 2024; Sama *et al*., 2017, 2018). However, EEE saponins are typically present as minor components in complex isomeric mixtures with high‐molecular‐weight triterpenoid saponins, which present hurdles to effective sourcing, purification, and thus their production. Interestingly, from an ecological perspective, some plants capable of synthesizing potent toxins, like ribosome‐inactivating proteins, also synthesize EEE saponins to synergistically increase their toxicity (Bolshakov *et al*., 2020; Melzig *et al*., 2005).

The recent elucidation of QS‐21's biosynthetic pathway marked a significant advancement in the understanding of plant enzymatic networks superseding the biosynthesis of this complex natural compound (Liu *et al*., 2024; Martin *et al*., 2024). This knowledge could potentially lead to improved production methods or the development of functional analogues such as the production of complex natural compounds in transgenic yeast (Liu *et al*., 2024).

Recently, our lab and others have been successful in the identification of enzymes involved in the biosynthesis of quillaic acid (QA) and its related saponins, such as saponarioside B from *S. officinalis*. These efforts elucidated the complete set of biosynthetic enzymes, including key cytochrome P450s catalysing specific oxidations at C‐23, C‐28, and C‐16 positions, as well as glycosyltransferases and acyltransferases critical for the decoration of the triterpene backbone with complex sugar moieties (Jo *et al*., 2024). Here, we report on the engineering of the biosynthetic pathway of SO1861, an EEE saponin from *S. officinalis* (Sama *et al*., 2017), in *G. elegans* (annual baby's‐breath or showy baby's‐breath), an ornamental plant native to Asia and Europe. Due to constraints in the genetic transformation of *S. officinalis*, we chose *G. elegans as a* closely related species known to synthesize SO1861 related compounds (Sama *et al*., 2018), and tested the possibility of heterologously expressing multiple genes to produce SO1861. Our results demonstrate that perturbing *CYP450* expression can potentially activate new, possibly latent, biosynthetic pathways, leading to the formation of novel compounds that are not typically produced by the plant's endogenous metabolism.

## Results

### Transcriptome analysis of *S. officinalis* identifies JA‐responsive saponin biosynthetic genes involved in SO1861 production

**Figure 1:**
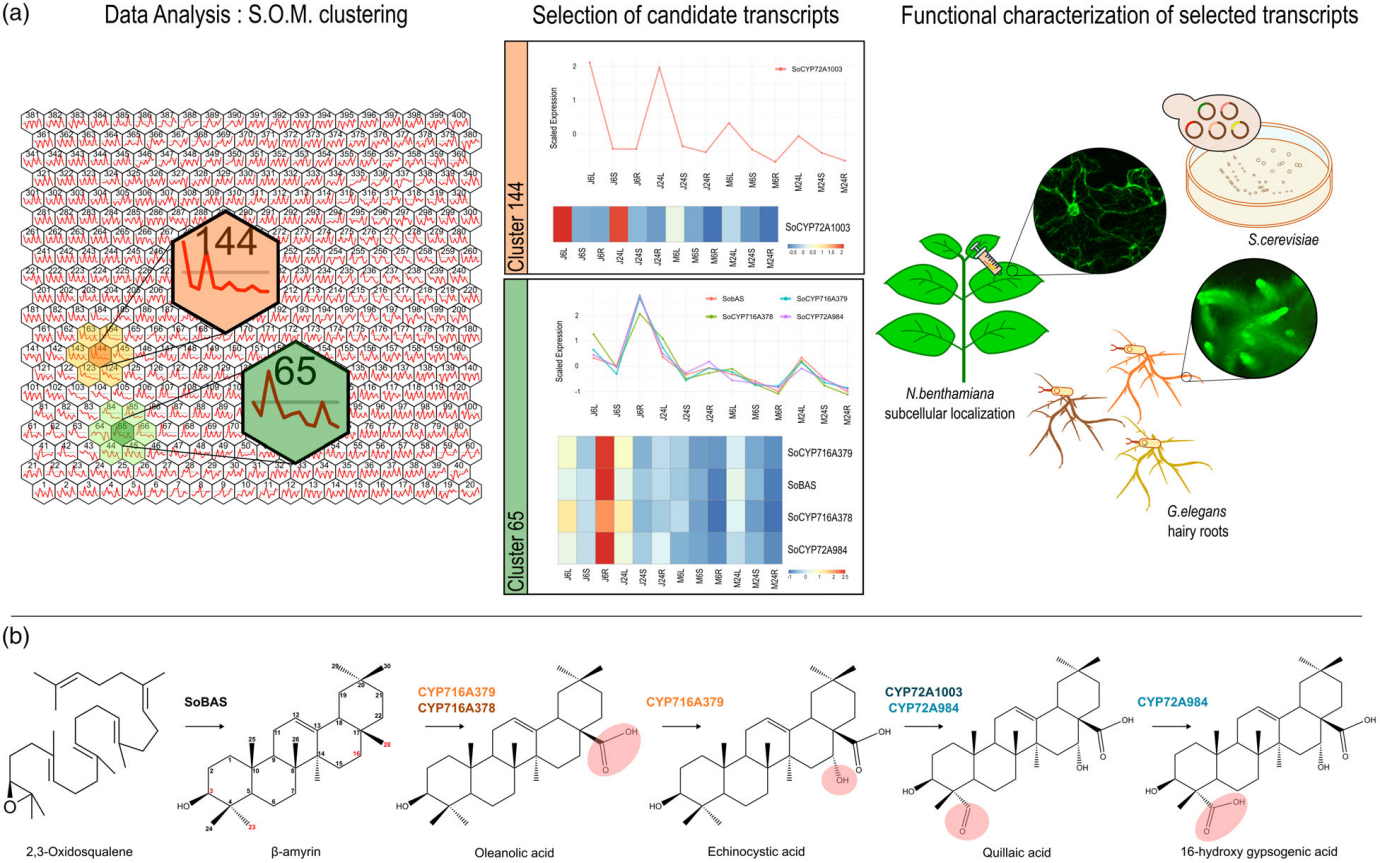
(a) Schematization of analysis and experimental workflow for identifying SO1861 biosynthetic enzymes in *Saponaria officinalis*. Gene expression data were analysed using Self‐Organizing Map (SOM) clustering (left panel). Cluster 65 (light green) and cluster 144 (light orange) are highlighted as containing key transcripts. Cluster 65 includes biosynthetic genes (*SoBAS*, *SoCYP716A378*, *SoCYP716A379*, *SoCYP72A984*), while cluster 144 contains *SoCYP72A1003*, the shorter C‐23 oxidase. Lighter shades highlight neighbouring clusters related to these two core clusters, with a representative expression trend (red line) shown for genes within each cluster. Center‐scaled expression values of selected genes from clusters 65 and 144 are presented as expression trends and heatmaps are shown in the middle panel (J = JA‐treated, M = mock, 6/24 = h after treatment, L = leaves, S = stems, R = roots), illustrating their transcriptional trend across different tissues and treatments. Highlighted genes from cluster 65 are predominantly expressed in JA‐elicited roots, peaking at 6 h post‐treatment, whereas *SoCYP72A1003* from cluster 144 shows peak expression in leaves at both 6 and 24 h post‐JA elicitation. Functional characterisation of selected transcripts was performed in heterologous systems (right panel), including *Nicotiana* leaves for subcellular localisation using GFP fusions, *Saccharomyces cerevisiae* for biochemical studies, and *G. elegans* hairy root cultures for further biochemical characterisation of oxidosqualene cyclase and cytochrome P450 enzymes. (b) Overview of key pathway steps from 2,3‐oxidosqualene to 16‐hydroxy gypsogenic acid, by *S. officinalis* cytochrome CYP450s identified in this study. Enzymatic steps catalysed by *S. officinalis* CYP450s are indicated in different colours; highlighted circles denote oxidized carbon positions.

To produce SO1861 in *G. elegans* hairy roots, we coupled gene discovery from *S. officinalis* with functional characterisation of candidate genes in yeasts and hairy roots (Figure 1). Multidimensional scaling (MDS) of the *S. officinalis* leaf, stem, and root transcriptomes showed that the main factor influencing the variation at the whole transcript level was explained by the type of tissue from which RNA was extracted (Figure S1). The first MDS dimension therefore explained 59% of the variation in the data and placed transcriptomes derived from *S. officinalis* stems between leaves and roots, thus mirroring the developmental gradient from underground to aerial parts of the plant. Another major driver of variation across the transcriptomic datasets was jasmonate (JA) treatment, accounting for 7% of variation at the whole transcript level. After filtering contigs displaying low expression or zero variance, data clustering was performed using the self‐organizing maps (SOM) algorithm (Orme *et al*., 2019; Payne *et al*., 2017; Wehrens and Buydens, 2007) to identify transcripts linked to SO1861 biosynthesis (Figure 1). This method has been reported to be particularly effective in clustering large datasets in an unbiased, unsupervised manner, allowing the identification of genes involved in the biosynthesis of complex plant specialized metabolites. The topology of the map was empirically optimized to accommodate on average 50 transcripts per node (i.e. gene cluster), balancing adequate grouping without excessive fragmentation. This step allowed for higher resolution over specific aspects of the biosynthetic pathway while avoiding the inclusion of distantly related transcripts/contigs, enabling the retrieval of meaningful data, even under low stringency conditions. Thus, each of the 400 SOM nodes (clusters) contained on average 50 transcripts. To identify SO1861 biosynthetic genes, the clusters were searched for the presence of a β‐amyrin synthase (Figure 1, SoBAS), the first gene responsible for the cyclization of the linear triterpene epoxy‐squalene to the pentacyclic triterpene β‐amyrin, the committed precursor for SO1861 production. Although the *S. officinalis* transcriptome assembly used to align the reads did not allow complete annotation of transcripts by TRAPID (Van Bel *et al*., 2013), the cluster containing the candidate SoBAS was found to harbour several transcripts encoding CYP450s and UDP‐glycosyltransferases; enzymes belonging to protein families involved in saponin production through oxidation and then glycosylation, respectively, of the triterpene carbon backbone (Figure 1, Figures S2, S3).

Transcripts grouped in the *SoBAS* cluster displayed highly dynamic expression trends across different tissues (leaves, stems, and roots), with a strong response to JA treatment. All the transcripts, including candidate *SoBAS* and the *CYP450s* selected for characterisation as involved in SO1861 production, showed a pronounced peak in expression under JA treatment, with the highest expression observed in JA‐treated roots (Figure 1, Figures S2, S3). This suggests that JA exerts a systemic effect on gene activation, with roots being particularly responsive. After this peak, expression levels generally decline significantly 24 h post‐treatment across all tissues, indicating a transient induction pattern. In non‐elicited conditions, transcripts of the selected genes were constitutively present in aerial parts, most likely because they participate in the biosynthesis of triterpenoid backbones for various saponins. This trend aligns with the well‐established role of JA responses in the temporal and spatial regulation of triterpenoid biosynthetic genes.

### *SoCYP450*s co‐expressed with *SoBAS* catalyse QA production

**Figure 2:**
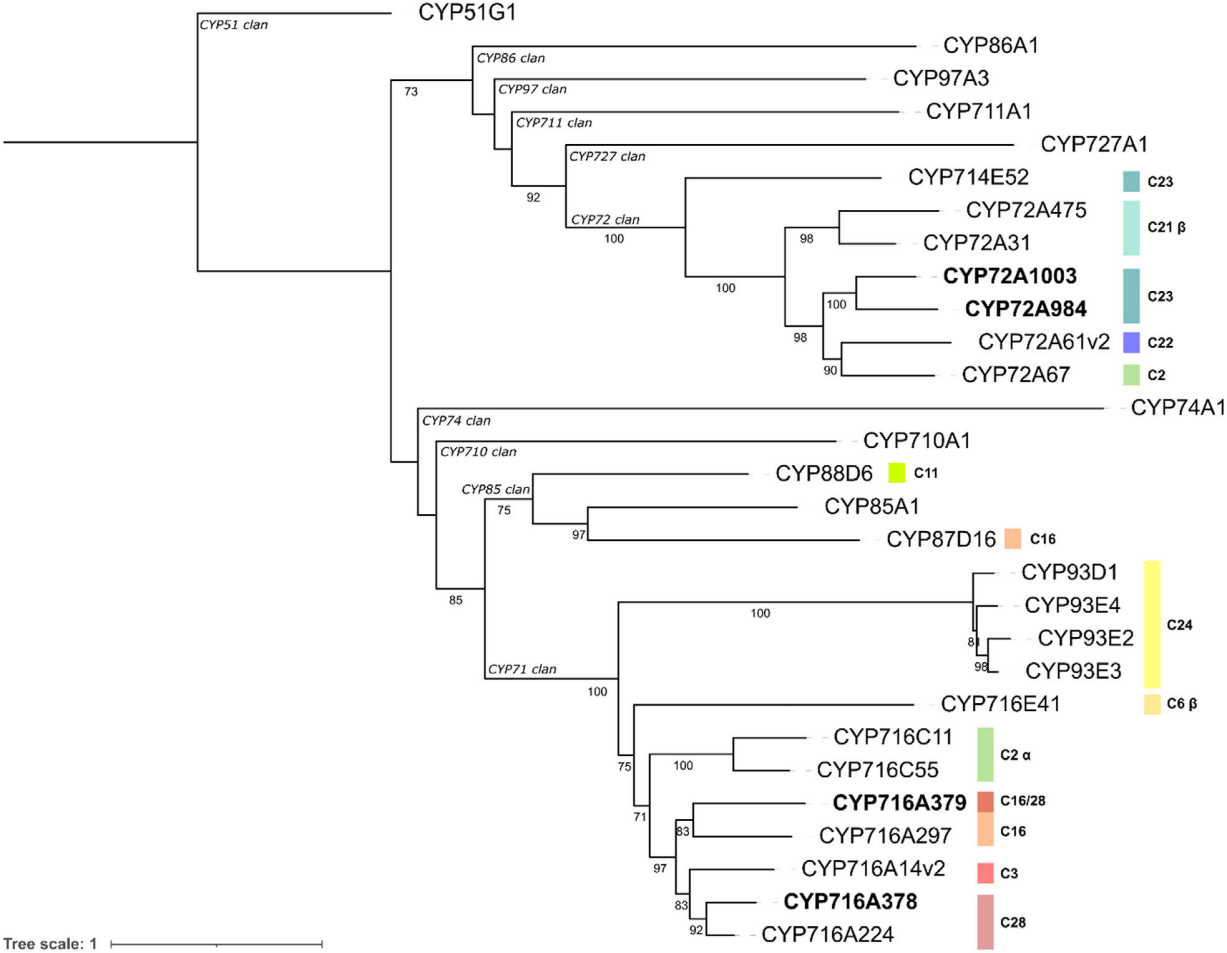
Phylogenetic analysis of plant cytochrome P450s (CYPs) in relation to those from *S. officinalis* identified in this study. The tree was generated using IQ‐TREE (v2.4.0) pipeline on usegalaxy.eu, based on translated protein sequences aligned with MUSCLE. ModelFinder was used to determine the best‐fit substitution model (LG + F + I + R4), and the maximum likelihood (ML) tree was inferred with 1000 ultrafast bootstrap replicates (UFBoot). Tree topology was refined using nearest‐neighbour interchanges (NNI), and branch lengths were recalculated accordingly. Sequences identified from *S. officinalis* are shown in bold. Bootstrap support values >70% are displayed at nodes along with P450 clan annotations as previously reported (Nelson and Werck‐Reichhart, 2011). Targeted carbon positions for CYP450s active on triterpenes are indicated at the right. The tree was rooted on CYP51G1.

Together with the candidate *SoBAS*, we identified three CYP450s whose assembled sequences were complete (Jo *et al*., 2024). Translated amino acid sequence alignment suggested that the three CYP450s shared homology with other known CYP450s that oxidize the oleanane backbone at C‐28, C‐16α/28 and C‐23 positions, hereafter referred to as SoCYP716A378, SoCYP716A379, and SoCYP72A984, respectively (Figure 2). Carboxylation at C‐28, aldehydelation at C‐23, and hydroxylation at C‐16α convert the triterpene β‐amyrin to QA, the SO1861 aglycone.

**Figure 3:**
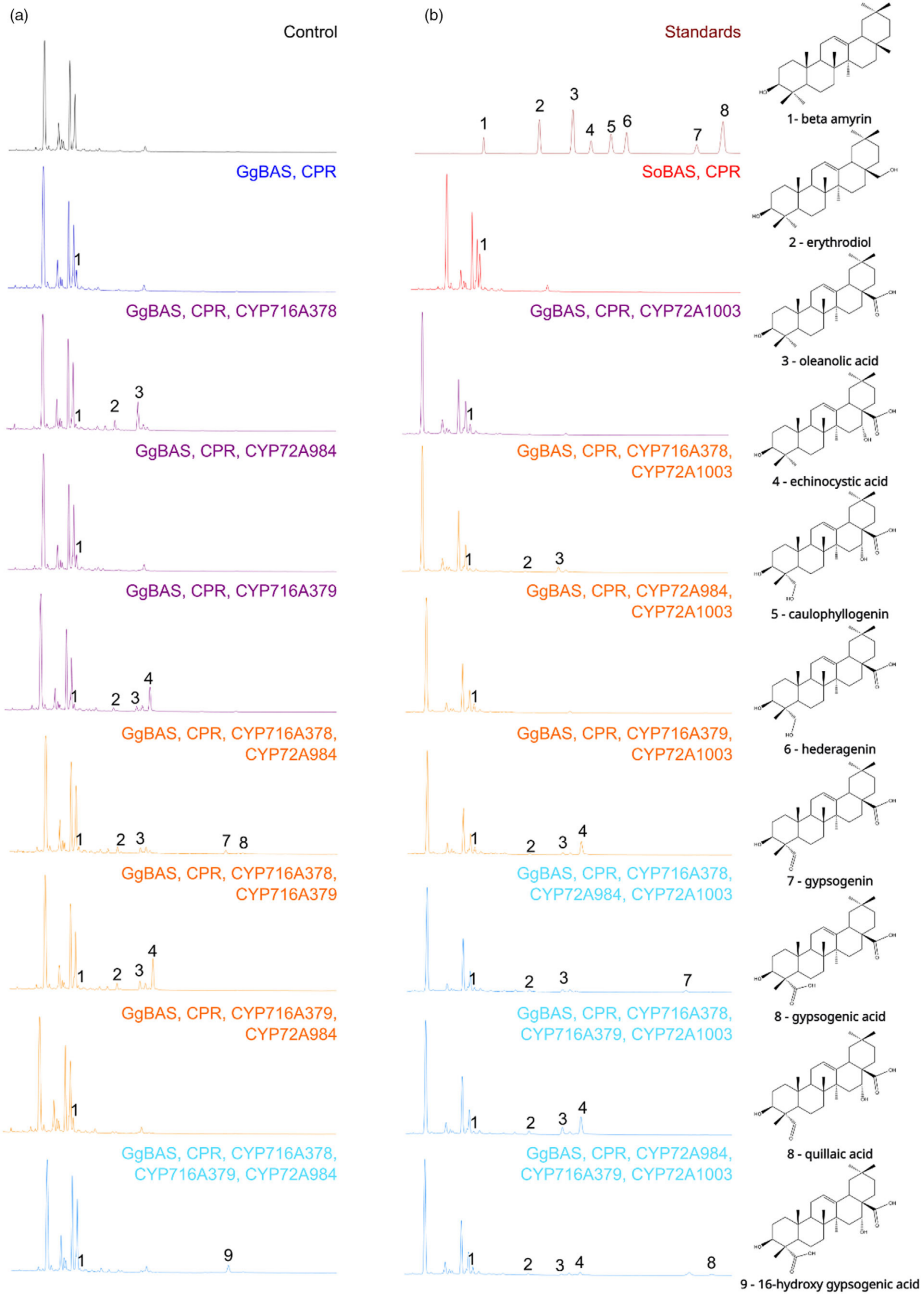
Functional characterisation of SoBAS, CYP716A378, CYP716A379, CYP72A984, and CYP72A1003 in yeast. All chromatograms in panel (a) and SoBAS, CPR in panel (b) show counts from 0 to 8.5E07 on the Y‐axis and acquisition time between 12 to 28 min on the X‐axis. Chromatograms containing CYP72A1003 and the standard mixture in panel (b) show counts from 0 to 3.8E07 on the Y‐axis and acquisition time between 12 to 28 min on the X‐axis. The standard mixture contains β‐amyrin (1), erythrodiol (2), oleanolic acid (3), echinocystic acid (4), caulophyllogenin (5), hederagenin (6), gypsogenin (7), gypsogenic acid and quillaic acid (8). Peak 9 corresponds to a triterpenoid for which the standard was not included and its corresponding electron impact (EI) mass fragmentation pattern is provided in Figure S18.

In Jo *et al*. (2024), we used transient plant expression assays in *Nicotiana benthamiana* leaves to demonstrate that SoCYP716A378, SoCYP716A379, and SoCYP72A984 indeed catalyse oxidation of β‐amyrin at the C‐28, C‐16α, and C‐23 positions, and function together to produce QA. Here, we performed a complementary functional characterisation of these CYP450s in the yeast *Saccharomyces cerevisiae*. To this end, the *S. officinalis CYP450* genes were cloned into high‐copy number plasmids suitable for yeast expression under the control of the galactose‐inducible promoter and with compatible auxotrophic markers for gene multiplexing. Yeast strains were obtained by transformation with individual saponin biosynthetic genes or in different combinations. The *SoCYP450* genes were expressed alongside partner enzymes essential for their activity or precursor biosynthesis. SoBAS was tested alone to confirm its functionality and specificity as a cyclase, while β‐amyrin synthase from *Glycyrrhiza glabra* (GgBAS – GenBank accession no. AB037203), which has been previously characterised (Hayashi *et al*., 2001), was included for use in concert with the SoCYP450s. Additionally, a feedback‐insensitive truncated version of *S. cerevisiae* 3‐hydroxy‐3‐methylglutaryl‐CoA reductase (tHMGR1) and the *Medicago truncatula* cytochrome P450 reductase (MtCPR1, Medtr3g100160) were co‐expressed to ensure a consistent flux through the metabolic pathway and to support efficient production of target compounds as previously reported (Moses *et al*., 2014; Tzin *et al*., 2019). Yeast liquid cultures were grown in induction media with galactose and methyl‐β‐cyclodextrin as a sequestering agent for the hydrophobic triterpene aglycones (Moses *et al*., 2014). The extracted spent media were analysed with gas chromatography–mass spectrometry (GC–MS) to identify the accumulating triterpene products (Moses *et al*., 2014). Yeast strains expressing *SoBAS* confirmed the presence of a peak corresponding to β‐amyrin (Figure 3, peak 1), matching those produced by strains expressing *GgBAS* and the analytical standard (Figure 3a, peak 1). In agreement with our previous report (Jo *et al*., 2024), co‐expression of *GgBAS*, *CPR,* and *SoCYP716A378* led to the accumulation of β‐amyrin and its C‐28‐oxidized derivatives, erythrodiol (C‐28‐OH) and oleanolic acid (C‐28‐COOH) (Figure 3a, peaks 1, 2 and 3). Similarly, when *SoCYP716A379* was expressed together with *GgBAS* and *CPR*, a new peak appeared, corresponding to echinocystic acid (C‐28‐COOH, C‐16α‐OH; Figure 3, peak 4), confirming that this enzyme also possesses a dual functionality, capable of oxidizing both C‐28 and C‐16α positions in yeast, as previously shown in agroinfiltrated *N. benthamiana* leaves (Jo *et al*., 2024). In contrast, however, no new peaks were detected in the yeast strain expressing the reported C‐23 oxidase *SoCYP72A984* alongside *GgBAS* and *CPR*. When testing co‐expression of *CYP450*s pairs with *GgBAS* and *CPR*, C‐28 oxidation products (erythrodiol and oleanolic acid) and C‐28/C‐23 oxidation products (gypsogenin and gypsogenic acid) were identified in lines co‐expressing *SoCYP716A378* and *SoCYP72A984* (Figure 3, peaks 2,3, 7 and 8), confirming the latter to be a C‐23‐specific CYP450. Although distinguishing between gypsogenic acid and QA via GC–MS is challenging due to their identical exact mass and retention times, the presence of gypsogenin (C‐28‐COOH, C‐23=O, Figure 3, peak 7) in the medium suggests that the second peak (Figure 3, peak 8) likely represents gypsogenic acid (C‐28‐COOH, C‐23‐COOH) rather than QA (C‐23=O, C‐16‐OH, C‐28‐COOH). Lastly, when these three *CYP450*s were co‐expressed, to our surprise, no peaks matching any of the analysed standards were identified, but a new peak was detected (Figure 3, peak 9). Mass spectrometry analysis revealed that the exact mass and fragment pattern of this peak were compatible with 16‐hydroxygypsogenic acid. Due to the lack of an analytical standard for this compound, yeast liquid cultures were scaled up to allow chromatographic purification of the peak for structural elucidation via nuclear magnetic resonance (NMR) (Figures S4–S7), which confirmed that the peak corresponded to 16‐hydroxygypsogenic acid (C‐28‐COOH, C‐16‐OH, C‐23‐COOH). Therefore, although these results confirmed the activity of all enzymes on the predicted carbon positions, their joint expression did not result in the production of QA in yeast, the triterpene that harbours a C‐23‐aldehyde. Instead, we found a triterpene bearing a C‐23‐carboxylic acid, suggesting further oxidation of the C‐23 position from the aldehyde to the carboxylic acid in the heterologous yeast system.

### Discovery of a non‐canonical SoCYP72 homologue that modulates C‐23 oxidation towards QA production in yeast cells

The genome size of *S. officinalis*, estimated via flow cytometry to range between 2.5 and 3.2 Gb (Figure S8), triggered us to assess its potential to harbour undiscovered *CYP450*s that were possibly overlooked in our RNA‐seq analysis or clustered outside of the node where most of our saponin biosynthetic genes were identified. Therefore, we performed a tblastn search against the *S. officinalis* contigs using the C‐23 oxidase SoCYP72A984 as the query. Notably, a new candidate CYP450 with high sequence homology but a shorter protein sequence compared to SoCYP72A984 was identified in a different SOM cluster (Figure 1, Figure S9).

In contrast to SoBAS and the three SoCYP450s reported so far, the transcript corresponding to the new candidate C‐23 oxidase, hereafter referred to as SoCYP72A1003 (Figure 2), displayed its highest expression in JA‐treated leaves at both 6 and 24 h (Figure 1, Figures S2 and S10), indicating a tissue‐specific response predominantly in aerial tissues. Consequently, *SoCYP72A1003* was cloned and its function tested in yeast for production of QA. Surprisingly, when *SoCYP72A1003* was co‐expressed with either the C‐28 oxidase (*SoCYP716A378*) or the C‐28/16α oxidase (*SoCYP716A379*), and the previously characterised C23 oxidase (*SoCYP72A984*), new peaks were observed corresponding to gypsogenin and gypsogenic/QA, respectively (Figure 3b, peaks 7 and 8). To unambiguously distinguish between gypsogenic acid and QA, liquid chromatography‐mass spectrometry (LC–MS) analysis was performed on metabolite extracts from the yeast strain expressing *GgBAS*, *CPR*, C‐28/16α oxidase (*SoCYP716A379*), and the two C‐23 oxidases (*SoCYP72A984* and *SoCYP72A1003*). The results confirmed that the simultaneous expression of these CYP450 enzymes led to the production of a peak matching the QA standard (Figure S11).

### Subcellular localisation in *N. benthamiana* leaves reveals the association of GFP‐tagged enzymes with the endoplasmic reticulum (ER) membrane

**Figure 4:**
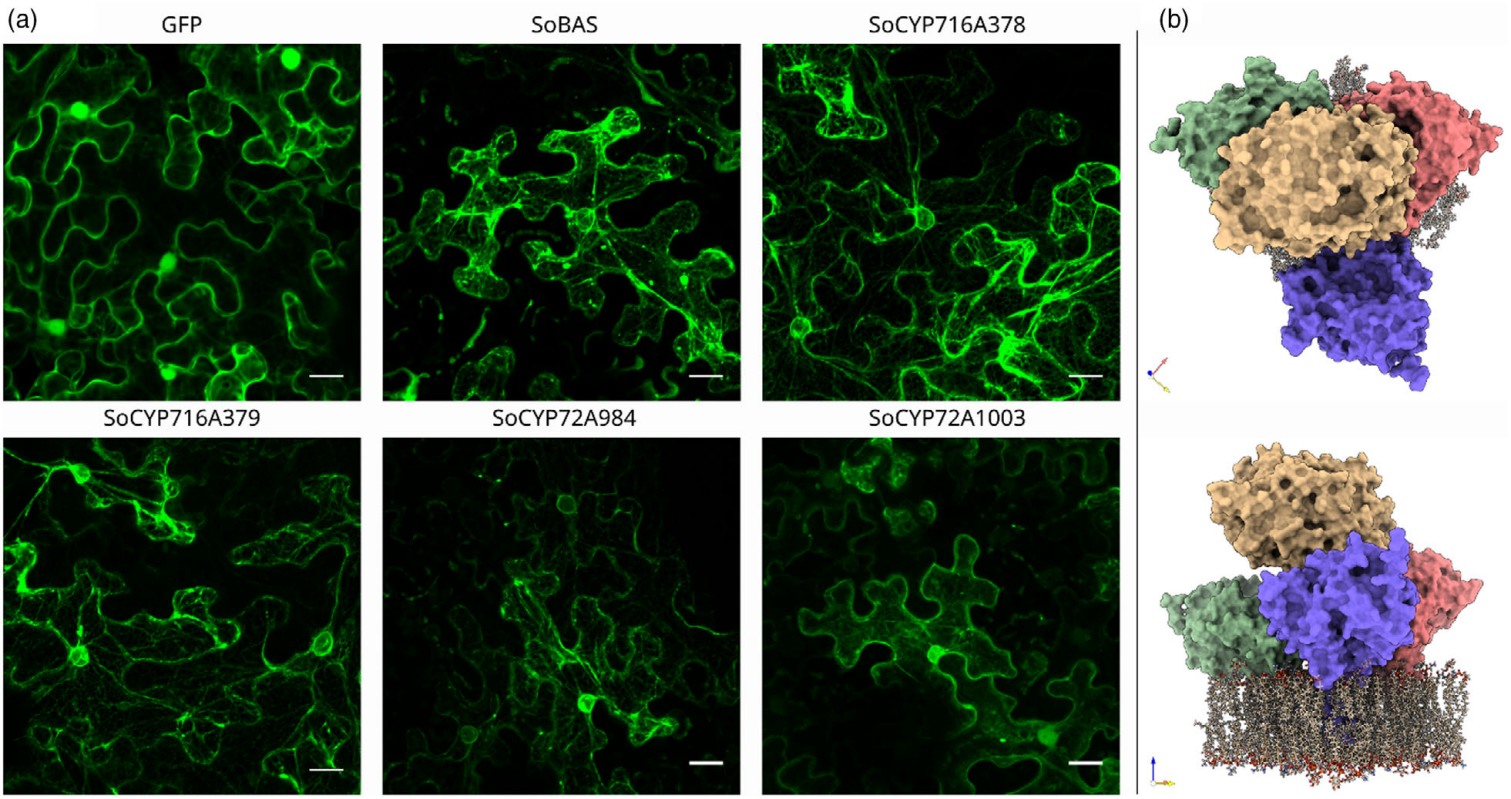
(a) Subcellular localisation of enzyme‐GFP fusions by laser scanner microscopy analysed in *Agrobacterium*‐infiltrated *Nicotiana benthamiana* leaves. Compared to the GFP control, all enzymes localize or are associated with the ER showing a typical net‐shaped pattern surrounding the nucleus. SoCYP72A1003 shows a more diffused localisation that includes the cytosol, typical of soluble proteins. As discussed, and presented in Figure S12, Alphafold3 modelling of its protein sequence revealed that this enzyme is missing the typical transmembrane domain that normally anchors CYP450s onto the surface of the ER. Scale bars = 20 μm. (b) Alphafold3 multimer model of a quaternary complex composed of SoCYP716A378 (purple), SoCYP716A379 (green), SoCYP72A984 (red), and SoBAS (yellow) inserted within the lipid‐bilayer of the plasma membrane (brown; lipid‐bilayer model available at CHARMM‐GUI Archive).

The ER and its surface are known to play a pivotal role in the biosynthesis of squalene‐derived triterpenes, as key enzymes involved in these pathways, such as the cyclases, CYP450s, and their associated partner CPR enzymes, are typically membrane‐bound (Arendt *et al*., 2017; Jaramillo‐Madrid *et al*., 2022). Although numerous studies have used colocalisation between tagged enzymes and fluorophores fused to ER‐retention signals, these methods often do not allow to fully visualise the intricate and extensive network of membranes and vesicles that permeate the intracellular space. To assess the structural features of the genes characterised in this study, Alphafold3 modelling was performed (Abramson *et al*., 2024). These models revealed that SoBAS as well as the C‐23 oxidase SoCYP72A1003 lacked a clear transmembrane domain canonically required for ER‐anchoring and localisation (Figure S12A,E). To investigate this further, we performed confocal microscopy localisation studies on C‐terminal GFP fusions of these enzymes expressed in agroinfiltrated *N. benthamiana* leaves. Compared to GFP controls, SoBAS, SoCYP716A378, SoCYP716A379, and SoCYP72A984 were clearly associated with the ER membrane because fluorescence signals for these four fusion proteins showed the net‐like spatial characteristic of the ER membrane network (Figure 4a). In contrast, the SoCYP72A1003‐GFP fusion protein displayed weaker ER association, visible by stronger fluorescence around the nucleus and in the cytosol of *N. benthamiana* cells. This observation could be explained by the fact that the hydrophobic membrane anchor domain, typically present at the N‐terminus of CYP450s, is missing. Therefore, it cannot be excluded that the SoCYP72A1003 sequence may be incomplete in our draft *S. officinalis* transcriptome. Having cloned this enzyme as a truncated version could make it soluble, but still partially able to physically interact with other membrane‐bound CYP450s through electrostatic interactions on the ER membrane. Similar interactions may also explain the ER localisation of SoBAS despite it lacking a transmembrane domain. In fact, physical proximity between CYP450s and partner enzymes has been proposed to impact their catalytic efficiency, although direct experimental evidence remains limited (Reed and Backes, 2017). To further support this hypothesis, we used Alphafold3 to model a quaternary complex between SoBAS, SoCYP716A378, SoCYP716A379, and SoCYP72A984. While being just a predictive model, the retrieved structure (Figure 4b) suggests that SoBAS may indeed interact with the membrane‐bound CYP450s, indicating a potential mechanism for the ER localisation of SoBAS.

### Constitutive expression of *SoCYP450* genes in *G. elegans* hairy roots enables heterologous production of SO1861

**Figure 5:**
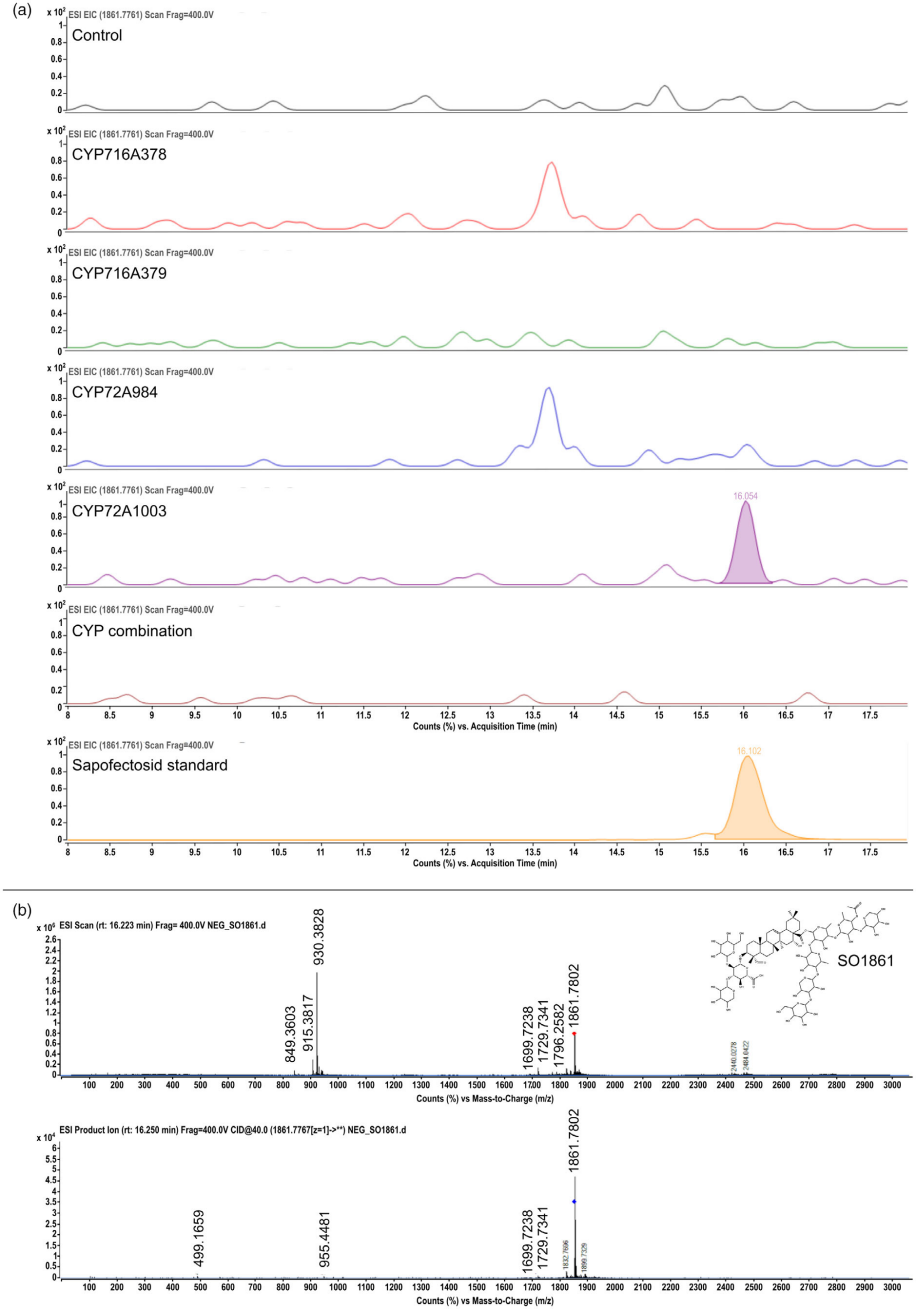
(a) Extracted ion chromatogram (EIC) of sapofectosid (SO1861, *m/z* 1861.7761) in transgenic *Gypsophila* hairy root lines expressing *GFP* as control, *CYP716A378*, *CYP716A379*, *CYP72A984*, *CYP72A1003*, or a combination of all *CYP450*s. Standard sapofectosid is plotted in orange. The EICs are scaled to the largest peak in each chromatogram and acquisition time between 8 to 18 min on the X‐axis. The sapofectosid peak is integrated when found in a hairy root line. (b) MS^1^ and MS^2^ fragmentation at 40 V collision energy of the sapofectosid peak eluting at 16.1 min. The SO1861 structure is reported on the top‐right corner.

To test how the activity of the newly identified SoCYP450s would impact the production of oleanane‐derived saponins, we overexpressed each of the four CYP450s individually or concomitantly with SoBAS in hairy roots. While we originally succeeded in generating *S. officinalis* hairy roots, they exhibited slow growth, weak transgene expression, and difficult cultivation (Figure S13), eventually hampering robust phenotyping. As a result, we opted to use hairy roots from another member of the Caryophyllaceae family, namely the ornamental plant *G. elegans*, which has been previously reported to produce triterpenoid saponins with EEE properties as well (Sama *et al*., 2018). Notably, one of the *G. elegans* saponins, known as gypsophilosid, shares high structural homology with *S. officinalis* SO1861, differing only by the substitution of an acetyl group for a xylose and by the presence of a xylose in place of a glucose as the last sugar moiety on the C28‐branched saccharide chain (Figure S14). Therefore, we hypothesised that *G. elegans* may have the enzymatic repertoire to produce SO1861‐related molecules, particularly as UDP‐glycosyltransferases acting on distant positions from the aglycone core often exhibit a certain degree of substrate promiscuity (Lacchini *et al*., 2023). Thus, we developed a hairy root transformation platform for this species. After the selection of *G. elegans* hairy roots based on GFP as a visible marker for transformation (Figure S13) and expression of *S. officinalis* enzymes (Figure S15), transgenic hairy root lines were grown in liquid culture for metabolite extraction and LC–MS profiling. The analysis showed that the expression of *SoCYP450*s could indeed modulate the saponin profile of *G. elegans* hairy roots. First, and rather unexpectedly, lines concomitantly expressing all enzymes together did not substantially differ from control GFP lines for most of the m/z values accounted for in the analysis (Figure 5a, Figure S16). That was also the case for lines only expressing *CYP716A378* or *CYP716A379* (Figure 5a, Figure S16). In contrast, both of the lines only expressing the C‐23 oxidases, *i.e*. either the canonical *SoCYP72A984* or the non‐canonical *SoCYP72A1003*, displayed higher abundance of peaks with MS/MS fragmentation patterns coherent with candidate precursors of gypsophilosid (Figure S16). Given that these precursors are shared with the *S. officinalis* EEE saponin SO1861, this was a promising finding. Surprisingly, however, only the lines expressing the non‐canonical *SoCYP72A1003* produced a peak matching the SO1861 standard (Figure 5). Conversely, in the lines expressing the canonical *SoCYP72A984*, an increased accumulation of gypsophilosid was observed (Figure S16). Taken together, these findings suggest (i) that C‐23 oxidation may represent a major metabolic constraint in the biosynthesis of QA‐derived saponins in *G. elegans*, (ii) that this constraint may be mitigated by co‐expression with non‐canonical *CYP450*s, and (iii) ultimately, that heterologous plant culture systems may be suitable production platforms for EEE saponin biosynthesis.

## Discussion

Our study highlights the potential of metabolic engineering to modulate the biosynthetic pathways of triterpenoid saponins, specifically focusing on saponariosides such as the EEE saponin SO1861 from *S. officinalis*. First, we started from our previously acquired *S. officinalis* saponarioside pathway knowledge (Jo *et al*., 2024) and assessed the functionality and specificity of the characterised SoBAS and the three SoCYP450s involved in the biosynthesis of the QA backbone (Jo *et al*., 2024) in the workhorse yeast *S. cerevisiae*. This confirmed their roles in catalysing key oxidation steps on the right carbon positions of the SO1861 precursor. Whereas the C‐28 oxidase SoCYP716A378 and the C‐28/16α oxidase SoCYP716A379 performed as expected, i.e. yielding the same products as when transiently expressed in *N. benthamiana* leaves (Jo *et al*., 2024), that was not the case for the C23‐oxidase SoCYP72A984. Indeed, co‐expression of *BAS* with these three *SoCYP450*s in yeast led to the accumulation of 16α‐hydroxygypsogenic acid, which has been reported as another triterpene backbone present in *S. officinalis* saponin extracts (Moniuszko‐Szajwaj *et al*., 2013). This compound differs from QA only by having a carboxyl moiety in place of an aldehyde moiety on C‐23. This may not be surprising, given that CYP450s have been reported to frequently catalyse sequential oxidations on the same carbon position going from hydroxy through the aldehyde to the carboxyl function (Bathe and Tissier, 2019; Carelli *et al*., 2011; Jo *et al*., 2024). Yet, it was remarkable that the sequential oxidation on C‐23 did not seem to occur that pronouncedly in *N. benthamiana* leaves.

Second, we speculated that the large *S. officinalis* genome could harbour more CYP450s involved in SO1861 biosynthesis, possibly one or some with a reduced proclivity to the second C‐23 oxidation. Indeed, additional blast searches identified such a CYP450 candidate. More precisely, we retrieved SoCYP72A1003, which corresponded to a shorter CYP450 that shared high similarity with the C‐23 oxidase SoCYP72A984 but had a different expression profile. TBLASTN with the SoCYP72A1003 amino acid sequence as the query against the core‐nucleotide_database within the Caryophyllaceae family on NCBI revealed our second C23 oxidase CYP72A984 as a closely related sequence within *S. officinalis* (Query Cover 99%, Identity 58.68%) and *Psammosilene tunicoides* CYP72A567 (Query Cover 99%, Identity 57.58%) as the second hit. When the search was extended to the Caryophyllales order, additional potential homologues could be found, including *Beta vulgaris* CYP72A397 (LOC104907331; 71.77% identity), *Spinacia oleracea* CYP72A397‐like (LOC110800618; 70.24% identity), and *Chenopodium quinoa* CYP72A15‐like (LOC110737899, 69.28% identity). Notably, co‐expression of *GgBAS*, *SoCYP716A379*, *SoCYP72A984*, and *SoCYP72A1003* led to the production of QA in yeast extracts, suggesting that substrate competition between the two C‐23 oxidases may allow the latter enzyme to accumulate detectable levels of QA. It has also been reported that differences in the affinity of plant UDP‐sugar transferases for QA rather than gypsogenic acid (GA) may influence saponin profiles by predominantly incorporating QA backbones. This sequestration of aldehyde‐bearing QA could prevent further sequential oxidation by CYP450, thus shaping the final composition of the saponin profile (Jo *et al*., 2024).

Subcellular localisation studies confirmed the ER as a critical site for triterpene biosynthesis as fluorescently tagged SoBAS and SoCYP450 enzymes were clearly localized in close association with the ER membrane. In contrast, SoCYP72A1003, which lacks a transmembrane domain, exhibited a weaker association with the ER, with fluorescence also observed in the cytosol. This suggests that SoCYP72A1003 might be partially soluble, likely due to the absence of a membrane anchor, yet it still retains some association with the ER. The weaker ER localisation could be explained by transient or indirect interactions with other ER‐bound enzymes, possibly through electrostatic interactions, which might affect its efficiency in the metabolic pathway. While in our case it has been a serendipitous finding, it is common practice in structural and functional studies on CYP450s to rely on truncation of the N‐terminus transmembrane domain in order to increase CYP450s' solubility while retaining their metabolic activity (Li and Poulos, 2004; Yamamoto *et al*., 2013). Alphafold3 predictive models were used to gather further support for the potential physical interaction occurring between saponin biosynthetic enzymes like SoBAS and the SoCYP450s, reinforcing the hypothesis that ER localisation, even without direct membrane attachment, plays a critical role in the efficiency and coordination of the biosynthetic pathways of complex saponins such as SO1861. By extension, this could apply to truncated SoCYP72A1003 as well.

Although hairy roots have long been known as a valuable system for genetic studies and as a platform for the production of plant‐specialized compounds with industrial applications (Murthy *et al*., 2017; Patra and Srivastava, 2018), so far there have been relatively few attempts at pathway engineering using this system (Chiyo *et al*., 2024). Given the structural and functional similarities—as EEE agent—between saponins from *G. elegans* and *S. officinalis* (Sama *et al*., 2017, 2018), we hypothesised that the former possessed the enzymatic machinery necessary to accommodate the production of SO1861‐related molecules. Therefore, by leveraging heterologous expression, we successfully expressed *SoCYP450*s in *G. elegans* hairy roots, being able to perturb a flux towards SO1861‐related compounds. The observed increases in candidate SO1861 precursors, particularly in lines expressing the C‐23 oxidase‐encoding *SoCYP72A984* and *SoCYP72A1003*, point to a critical role of C‐23 oxidation (Takahashi *et al*., 2016) in the biosynthesis of QA‐derived saponins as likely the last step prior to the commitment of aglycones to the glycosylating pathway (Jo *et al*., 2024).

Interestingly, the simultaneous overexpression of all four *CYP450*s did not significantly alter the saponin profile compared to the control GFP line, raising the possibility that concomitant expression of several enzymes, some of which will have a redundant function with endogenous *G. elegans* enzymes, may simply feed into the existing saponin biosynthetic pathways without altering the overall composition. It is likely that combining overexpression with CRISPR‐mediated knockout of endogenous competing pathways could be more effective in directing substrates towards specific products (Chiyo *et al*., 2024). For instance, the metabolic pathways of plant specialized metabolites are far from linear but better represented by complex networks of enzymes that, with different affinities and abundances, produce and compete for precursors, thus greatly expanding the chemical diversity of the final output (Ji *et al*., 2024).

The observation that lines expressing the truncated non‐canonical C‐23 oxidase SoCYP72A1003 produced a peak matching the SO1861 standard is particularly intriguing and suggests that even partial enzyme truncations may retain or, in certain contexts, even enhance a desired catalytic activity. Furthermore, since SoCYP72A1003 transcripts, differently from those encoding the previously characterised enzymes from *S. officinalis* saponarioside biosynthesis, exhibited a JA response predominantly in aerial tissues rather than in roots, it also implies that pathways potentially could be engineered by simply perturbing the spatial expression profile of endogenous enzymes. This mix‐and‐match approach of novel enzymatic combinations could thus lead to the production of new compounds. We also speculate that species‐specific saponin profiles may be dictated and constrained by physical association between triterpene biosynthetic enzymes that, when perturbed, could open new, previously latent, metabolic pathways through the action of promiscuous enzymes, such as UDP‐glucuronosyltransferases, leading to novel metabolic products.

## Materials and methods

### RNA extraction, sequencing, transcriptome assembly, analysis, and selection of candidate genes

To identify the genes encoding the enzymes involved in the biosynthesis of triterpenoid saponins in *S. officinalis*, a draft reference transcriptome was built using a publicly available dataset through the 1KP project (One Thousand Plant Transcriptomes Initiative, 2019) and derived from HiSeq paired‐end sequencing of flowers, fruits, stems, and leaves assembled using de novo transcriptome assembly on the QIAGEN CLC Genomics Workbench using default parameters. The resulting contigs were annotated via the web‐based platform TRAPID (Van Bel *et al*., 2013). The co‐expression analysis was performed using data specifically generated for this study. RNA expression datasets comprised different organs sampled in response to hormonal treatments at two time points. For this purpose, leaves, stems, and roots from 3‐month‐old *S. officinalis* plants grown in hydroponics were sampled in triplicate at 6 and 24 h after mock or 50‐μM methyl‐jasmonate treatment, for a total of 36 RNA samples. Each of the three biological replicates is a pool of tissues from three different plants. *S. officinalis* seeds were sourced from the seed company Jelitto (https://www.jelitto.com/, Schwarmstedt, Germany). To this end, RNA was extracted from the sampled *S. officinalis* tissues using the ReliaPrep RNA miniprep Systems (Promega™, Madison, WI, USA), following the manufacturer's instructions for fibrous tissues. RNA was single‐end sequenced via Illumina HiSeq 6000 with single‐end read lengths of 100 bp for gene expression profiling. RNA was also used as a template for cDNA synthesis using qScript cDNA SuperMix (QuantaBio™, Beverly, MA, USA) and for the cloning of candidate genes. Transcript levels were estimated by mapping single‐end reads on the S. officinalis transcriptome using Salmon (Patro *et al*., 2017) implemented on a Galaxy pipeline. Next, the analysis was performed in RStudio. After count per million (CPM) normalisation, transcripts with zero expression or variance across samples as well as those lowly expressed (≤5 CPM in 6 of the 36 datasets) were removed. Following this, TMM (trimmed mean of M‐values) normalisation was applied to account for library size differences. The remaining genes were clustered using the self‐organizing map (SOM) algorithm implemented and visualised in R using the Kohonen package (Wehrens and Buydens, 2007) to assemble clusters of transcripts characterised by similar expression trends. Transcripts‐scaled expression values were mapped on a 20 × 0 hexagonal grid (400 nodes) with toroidal topology, training the algorithm for 350 iterations, aiming at about 50 transcripts per cluster. Reverse Transcription Quantitative PCR (RT‐qPCR) experiments were performed using the same RNA extraction protocol and cDNA kit as reported above.

### Phylogenetic analysis of plant CYP450s

Protein sequences were downloaded from UniProt, aligned using MUSCLE, and phylogenetic analysis was carried out using IQ‐TREE (v2.4.0) (Minh *et al*., 2020) on usegalaxy.eu. Model selection was performed using ModelFinder (Kalyaanamoorthy *et al*., 2017) to identify a best‐fit substitution model. Maximum likelihood (ML) tree inference was conducted incorporating both ModelFinder and tree reconstruction. Branch support was assessed using ultrafast bootstrap (UFBoot) (Hoang *et al*., 2017) with 1000 replicates. Tree topology was optimized using a combination of nearest‐neighbour interchanges (NNI) and branch length recalculation. The resulting tree was visualised using iTOL v7.

### Cloning of candidate genes from *S. officinalis* and generation of expression constructs for functional analysis

Each target gene described above was amplified by PCR using specific primer sets (Table S1) from *S. officinalis* cDNA. Each primer included adapter sequences at the 5′ end to allow directional cloning in appropriate vectors. For each gene, the PCR product was gel‐purified and recombined into the Gateway entry vector pDONR221, enabling downstream applications using Gateway‐based destination vectors. These included expression in yeast and the generation of *G. elegans* hairy roots expressing single genes. Differently, the PCR product was recombined into the pGGC000 Golden Gate‐compatible vector (Vector ID: 17_71, available at https://vectorvault.vib.be/) for the assembly of transcriptional cassettes designed for multiplexed gene expression in hairy roots. Golden Gate‐compatible modules (available at https://vectorvault.vib.be/) were also used to produce C‐term translational GFP fusions (Vector ID: 13_62, https://vectorvault.vib.be/) for localisation studies in *Nicotiana* agroinfiltrated leaves. The resulting plasmids were transformed into *E. coli* DH5a, extracted and sequenced to verify the presence of correct inserts before proceeding with yeast or *Agrobacterium* transformation.

### Subcellular localisation of *S. officinalis* triterpene biosynthetic enzymes in *N. Benthamiana* leaves

The subcellular localisation of *S. officinalis* triterpene biosynthetic enzymes was performed using translational GFP fusions via *Agrobacterium*‐mediated transient expression in *N. benthamiana* leaf epidermal cells. The coding sequences of *SoBAS* and *SoCYP450*s were expressed under the control of the 35S promoter and fused in frame with eGFP at their C‐terminus. Golden Gate‐compatible vectors from the VIB Vector Vault were used to generate these constructs. For the eGFP control, Gateway‐binary vector pK7WG2D was used. Each binary vector was transformed into the *A. tumefaciens* strain C58C1, and bacterial suspensions with an optical density (OD600) of 0.3 were used for leaf infiltration.

72 h post‐infiltration, leaf sections were harvested and analysed for fluorescence using a Zeiss LSM710 laser scanning confocal microscope equipped with a Plan‐Apochromat 20x/0.8 M27 objective. GFP was excited at 488 nm with an Argon laser, and Z‐sections were captured at 2‐μm intervals. The resulting images were processed to create maximum intensity projections, with scale bars added using Fiji software.

### Protein modelling of SoBAS and the SoCYP450 complex

The tetrameric protein complex of SoBAS and the three SoCYP450s was modelled using AlphaFold3. Protein complex orientation within the membrane was analysed using the PPM 3.0 Web Server. A plant‐specific membrane model was downloaded from CHARMM‐GUI (Jo *et al*., 2008). ChimeraX was employed to assemble and visualise the protein‐membrane system.

### Flow cytometry for *S. officinalis* genome size estimation

Young leaves of *S. officinalis* were finely chopped using a razor blade in 200 μL of CyStain® UV Precise nuclei extraction buffer (Sysmex, Norderstedt, Germany). To stain DNA, 800 μL of staining buffer was added. After a quick spin to pellet debris, the suspension containing nuclei was analysed using a CyFlow Flow Cytometer (Partec, Goerlitz, Germany). To estimate the *S. officinalis* genome size, samples were spiked with nuclei extracted from *Arabidopsis thaliana* seedlings or from maize (*cv*. B73) as references.

### Constructs for *G. elegans* hairy root transformation

*Gypsophila elegans* seeds were sourced from the seed company B&T World Seeds (Paguignan, France). Hairy root transformation of *G. elegans* via *Agrobacterium rhizogenes* was conducted following a previously described protocol for *Medicago truncatula* with slight modifications (Pollier *et al*., 2011). Seeds were sterilised using a 4% sodium hypochlorite solution for 20 min and then thoroughly rinsed with sterile water. The sterilised seeds were germinated on Murashige and Skoog (MS) medium containing 1.5% sucrose (pH 5.8) under long‐day conditions (16 h of light) at 24°C. After 10 days of growth, the radicles of germinated seedlings were wounded by removing approximately 4 mm from the root tip. These wounded seedlings were inoculated with *A. rhizogenes* strain LBA9402 carrying appropriate binary vectors and placed on inclined agar squared plates with MS medium (pH 5.8) supplemented with vitamins (Duchefa, Haarlem, The Netherlands). Plates were sealed with micropore tape and incubated vertically for 10 days under the same conditions for cocultivation.

Following the cocultivation period, seedlings were transferred to fresh plates containing MS medium supplemented with 100 mg/L cefotaxime to inhibit the growth of *A. rhizogenes*. Plates were placed vertically under long‐day conditions at 24°C. After 30 days, *G. elegans* hairy roots, expressing *eGFP* as a transformation marker, were selected, excised, and transferred to rounded petri solid MS medium (pH 5.8) containing vitamins, 3% sucrose (w/v), 8% Plant Agar, and 100 mg/L cefotaxime to ensure the elimination of any bacterial contamination. The plates were sealed and maintained in the dark at 24°C. After 3 weeks, young hairy root tissues were transferred onto solid MS medium without antibiotics. To maintain the cultures, hairy roots were subcultured and transferred to fresh plates every 4 weeks. For metabolic profiling, the hairy roots were subcultured from solid medium and grown for 30 days in liquid MS medium (pH 5.8) with vitamins and 3% sucrose (w/v).

For constitutive expression of individual genes, coding sequences were individually cloned into the Gateway‐compatible pK7WG2D binary vector under control of 35S. The vector also contains *eGFP* under control of the *RolD* promoter as a visible marker for transformation. The same empty vector, only containing *eGFP*, was used to produce control hairy root lines. Differently, concomitant expression of multiple genes was achieved by modular Golden Gate assembly of different independent transcriptional units (promoter, CDS, terminator) into intermediate Gibson‐compatible entry vectors. In this vector, each transcriptional module (i.e. promoter‐gene‐terminator) is flanked by a unique nucleotide sequence designed to be compatible with Gibson assembly. Upon I‐SceI digestion, these modules were seamlessly assembled into a single binary vector pKU1U9_ccdB containing all transcriptional cassettes (Vector ID: 11_43, available at https://vectorvault.vib.be/). The resulting binary vector contained several transcriptional cassettes for concomitant expression of *SoBAS*, *SoCYP716A378*, *SoCYP716A379*, *SoCYP72A984*, *SoCYP72A1003*, and *eGFP*. To minimize the chance of silencing, four different constitutive promoters, available at the VIB collection, were chosen to drive the expression of seven coding sequences as schematized in Figure S17.

### RT‐qPCR analysis

RT‐qPCR was conducted using the LightCycler 480 system (Roche, Basel, Switzerland), SYBR Green Master Mix (Thermo Fisher Scientific, Waltham, MA, USA), and primers (Table S1) designed with NCBI primer blast. The reactions were carried out in 384‐well plates with a total volume of 5 μL per reaction using a Tecan. The protocol included an initial denaturation step at 95°C for 5 min, followed by 45 PCR cycles comprising denaturation at 95°C for 10 s, annealing at 60°C for 15 s, and extension at 72°C for 15 s. Reactions were done in triplicate using *G. elegans UBIQUITIN 10* as the reference gene.

### Heterologous expression in yeast

For heterologous expression in yeast, five different expression vectors were selected that (i) allow inducible transgene expression upon switching from glucose to galactose as the carbon source in the culture medium and (ii) carry different auxotrophic markers. Therefore, SoBAS_1421 was cloned into pESC (uracil selection). This vector was modified to be used in Gateway recombination systems, as described previously (Miettinen *et al*., 2017). The same plasmid also contained a feedback‐insensitive truncated version of 3‐hydroxy‐3‐methylglutaryl coenzyme A reductase 1 (tHMGR1) from *Medicago truncatula*. *tHMGR* genes have been extensively reported to increase triterpene production upon transient or stable expression in heterologous as well as homologous systems (Holmberg *et al*., 2003). *SoCYP450_1944*, *SoCYP450_2010*, and *SoCYP450_1049* were cloned into the Gateway‐compatible yeast vectors pAG424GAL (tryptophan selection), pAG425GAL (leucine selection), and pAG427GAL (methionine selection), respectively. *SoCYP450_6085* was cloned in all three different vectors (pAG424GAL, pAG425GAL, and pAG427GAL) to allow combination with each of the other CYP450s. Finally, in order to increase the catalytic efficiency of the CYP450s, a partner reductase enzyme from *M. truncatula* (MtMTR1 – MTR_3g100160) was cloned into pAG423GAL (histidine selection). All vectors contain galactose‐inducible promoters driving the expression of inserted genes.

All these plasmids were transformed in *S. cerevisiae* to assess the function of each gene individually or in different combinations, as described in Table S2. The yeast strain used in this study was derived from BY4272 (genotype: MATa; his3Δ1; leu2Δ0; ura3Δ0; lys2Δ0; trp1Δ0; met15Δ0; PAH1‐0b; Perg7::PMET3‐ERG7), which contains five auxotrophic selection markers (‐URA/‐HIS/‐LEU/‐MET/‐TRP) and therefore allows expression of genes from up to five plasmids.

The transformed yeast strains were selected on solid synthetic defined yeast media with appropriate supplements. Selected yeast strains were cultured in synthetic liquid medium with galactose as the only carbon source and incubated for 7 days at 30°C. Methyl‐β‐cyclodextrins were added to the liquid medium at day two and day four during cultivation to reach a final concentration of 10 mM. Methyl‐β‐cyclodextrins are cyclic oligosaccharides that are able to sequester apolar triterpenes from yeast cells into the liquid medium, thus avoiding possible toxicity and feedback inhibition by pathway intermediates, and thereby increasing their production yield (Moses *et al*., 2014). Yeast cultures were pelleted by centrifugation and metabolites were extracted separately for cell pellets and liquid culture medium by liquid–liquid separation using n‐hexane and ethyl acetate as previously reported (Moses *et al*., 2014).

### Gas chromatography–mass spectrometry analysis

The triterpene profiles were analysed on an Agilent 7890B gas chromatogram (GC) coupled to an Agilent 7200B quadrupole time‐of‐flight mass spectrometer (QTOF‐MS) with GERSTEL multipurpose sampler (MPS) robotics (Element). The dry samples were derivatized with 50 μL pyridine: N‐methyl‐N‐trimethylsilyltrifluoroacetamide (1:4) by incubation at 37°C, 750 rpm for 1 h. Trimethylsilylated samples (1 μL) were injected at a split ratio of 10:1, with a split flow of 10 mL/min into a DB‐5 ms 40 m × 250 μm × 0.25 μm GC column (Agilent Technologies, Santa Clara, CA, USA). Helium was used as the carrier gas at a flow rate of 1 mL/min, the inlet was set to 250°C and the GC oven was programmed to 100°C for 1 min, followed by ramping at 60°C/min to 300°C, where it was held for 25 min. The ion source was set to 230°C, 35 μA filament current, 70 eV electron energy and the mass range of 50–1000 m/z was scanned at an acquisition rate of 4 spectra/s with a solvent delay of 5 min. Total ion chromatograms and mass spectra were analysed using the Agilent MassHunter Qualitative Analysis B.10.00 software.

### Liquid chromatography–mass spectrometry analysis

The saponin profiles were analysed using liquid chromatography (LC coupled to ion mobility (IM) quadrupole time‐of‐flight (qTOF) mass spectrometry (MS)). The instrumentation consisted of an Agilent 1290 Infinity II series UHPLC system hyphenated with an Agilent 6560 IM‐qTOF with a Dual Agilent Jet Stream Electron Ionization source. LC separation was performed on a ZORBAX Extend‐C18 column (Agilent Technologies 727 700‐902). A 29‐min gradient was run using the organic buffer B (acetonitrile, 0.1% formate) combined with the aqueous buffer A (water, 0.1% formate). Data was acquired using MassHunter Data Acquisition 10.0 software on 5 μL of sample separated on the column with a flow rate of 300 μL/min. The LC gradient consisted of 99% buffer A at min 0, 50% buffer A at min 20, 30% buffer A at min 25, 0% buffer A at min 27, and returned to 99% buffer A at min 28, where it was maintained for another minute. Data was acquired in negative ionization mode between the 50 to 3200 m/z range, with an MS acquisition rate of 1 spectra/s and MS/MS acquisition rate of 3 spectra/s. A fixed collision energy of 40 V was used with a maximum of 2 precursors per cycle. The raw data files were visualised using the Agilent MassHunter Qualitative Analysis B.10.00 software and extracted ion chromatograms were obtained from MS level 1 scan with symmetric m/z ± 0.5.

### Isolation of 16‐OH‐GA from yeast medium for NMR‐based structure elucidation

Methyl‐β‐cyclodextrin‐treated yeast spent medium was subjected to high‐performance liquid chromatography‐mass spectrometry (HPLC‐MS) at the VIB Metabolomics Core Ghent. 10 mL was injected on an LC Prep AutoPurification System (Waters, Milford, MA, USA) connected to an Acquity QDa Mass Detector (Waters). Chromatographic separation was carried out on an XSelect HSS T3 OBD Prep Column (10 × 150 mm; 5 μm; 100 Å) (Waters); the column temperature was maintained at room temperature. A gradient of two buffers was used for separation: buffer A (water +0.1% formic acid, pH 3) and buffer B (acetonitrile +0.1% formic acid, pH 3), as follows: 99% A decreased to 50% A in 30 min, decreased to 30% from 30 to 35 min, and decreased to 0% from 35 to 37 min. The flow rate was set to 10 mL/min. Post chromatographic separation, the flow and a make‐up buffer (75% water +25% acetonitril +0.1% formic acid, pH 3) of 1 mL/min were fed into a 5000:1 flow splitter. The lowest flow was subjected to Electrospray Ionization (ESI) under the following specific conditions: capillary voltage, 0.8 kV; source temperature, 120°C; desolvation gas temperature, 600°C. Mass range was set from 100 to 1200 Da for function 1; a Single Ion Recording (SIR) with mass 501.32 Da was set for function 2; scan time was set at 1 Hz; nitrogen (>96%) was employed as desolvation and cone gas. Centroid data was recorded through MassLynx V4.1 (Waters). Peak detection parameters used for collection were set to: target mass, 501.3 Da; mass window, ±0.5 Da; Minimum Intensity Threshold (MIT), 500 counts; minimum peak width, 3 s; solvent delay towards the highest flow, 26 s. CentriVap Benchtop Vacuum Concentrator (Labconco) was used to evaporate collected fraction until dry.

### Nuclear magnetic resonance spectroscopy

The lyophilized sample was dissolved in CD3OD (99.96% purity). All NMR spectra were acquired at 298 K on a Bruker Avance III HD 800 MHz spectrometer, equipped with a TCI cryoprobe for enhanced sensitivity. The experimental set comprised 1D ^1^H (64 k data points, relaxation delay d1 = 1 s, spectral width SW = 20 ppm, transmitter frequency offset O1P = 6.175 ppm), 2D ^1^H‐^13^C HSQC (1 k and 256 data points in t2 and t1, respectively, relaxation delay d1 = 1.5 s, spectral widths SW of 13 ppm in t2 and 150 ppm in t1, offsets of 6.175 ppm in t2 and 55.0 ppm in t1, apodization with sine bell (SSB = 2) in both dimensions, zero‐filling with linear prediction up to 1 k in t1), and 2D HMBC spectra (4 k and 256 data points in t2 and t1, respectively, relaxation delay d1 = 2 s, low‐pass J‐filter corresponding to 1 J(^1^H‐^13^C) = 145 Hz, spectral widths SW of 13 ppm in t2 and 200 ppm in t1, offsets of 5.500 ppm in t2 and 90.0 ppm in t1, apodization with sine bell (SSB = 2) in both dimensions, zero‐filling with linear prediction up to 1 k in t1, data displayed in magnitude mode with xf2m processing command). The NMR data were acquired and processed in TopSpin 3.6 (Bruker); the chemical shifts were referenced to the residual solvent signals at 3.310 ppm (^1^H) and 49.15 ppm (^13^C). Despite a limited amount of material and the presence of contaminants giving rise to strong NMR signals (Figure S5), we obtained complete assignments of carbon atoms and carbon‐bound protons of the metabolite, which allowed its unequivocal identification as 16α‐hydroxygypsogenic acid (Figure S4). Aided by published NMR analyses of pentacyclic triterpenes (Gevrenova *et al*., 2006; Mahato and Kundu, 1994; Rodríguez‐Díaz *et al*., 2011) and starting from the stereochemistry of β‐amyrin scaffold, we have elucidated the structure of 16α‐hydroxygypsogenic acid in several steps. First, based on their typical chemical shifts (for instance, observed in the QA (Rodríguez‐Díaz *et al*., 2011)), we readily identified the olefinic CH group (position 12, δ_H_ = 5.30 ppm, δ_c_ = 123.3 ppm), and two CH‐OH moieties (position 3, δ_H_ = 3.97 ppm, δ_c_ = 76.6 ppm; and position 16, δ_H_ = 4.46 ppm, δ_c_ = 75.4 ppm). Then, following their correlations in the HMBC spectrum (Figure S7), we assigned a number of neighbouring atoms, which in turn could be connected to the rest of the molecule via the sequential HMBC “walk”. Finally, we relied on the published data (Gevrenova *et al*., 2006) to disambiguate the assignments of methyl groups at positions 29 and 30. The full NMR analyses are presented in Figures S4–S7.

## Conflicts of interest

The authors declare no competing interests.

## Author contributions

A.G. initiated, the project, and wrote the manuscript; E.L. designed and performed experiments, analysed data, and wrote the manuscript; T.Q., T.M., and O.E. designed and performed experiments and analysed data; all authors discussed the results and commented on the manuscript.

## Supporting information


**Table S1.** Primers used for gene cloning and expression analysis.

**Table S2.** Summary of engineered yeast strains generated in this study, including the expressed saponin biosynthetic genes, corresponding plasmid vectors, and triterpene aglycones detected through metabolite analysis.

**Figure S1.** Global transcriptomic changes visualised by multidimensional scaling (MDS), showing gene expression variance between JA‐treated (bottom‐half) and mock‐treated (top half) samples, as well as across tissues (leaves, stems, roots).
**Figure S2** Count per millions (CPM) expression values in *Saponaria* RNA‐seq data for all candidate gene transcripts considered in this study across different tissues.
**Figure S3** Heatmap reporting scaled expression values (mean‐centered) from *Saponaria* RNA‐seq data of the SOM cluster 65 (i.e. node) containing *SoBAS* and *SoCYP450s* characterised in this study (in bold).
**Figure S4** Summary of the NMR analysis of the samples containing 16α‐hydroxygypsogenic acid.
**Figure S5** ^1^H NMR spectrum of the sample containing 16α‐hydroxygypsogenic acid.
**Figure S6** ^1^H‐^13^C HSQC spectrum of the sample containing 16α‐hydroxy‐gypsogenic acid.
**Figure S7** HMBC spectrum of the sample containing 16α‐hydroxy‐gypsogenic acid.
**Figure S8** Flow cytrometry of DAPI‐stained nuclei from *Arabidopsis*, maize and *S. officinalis*.
**Figure S9** ClustalW comparison of protein sequences of the *Saponaria* CYP450s identified in this study.
**Figure S10** Heatmap reporting scaled expression values (mean‐centered) from *Saponaria* RNA‐seq data of the SOM cluster 144 (i.e. node) containing *SoCYP72A1003* (in bold). Rows represent gene transcripts and columns represent experimental tissues/treatments.
**Figure S11** LC‐MS XIC chromatograms comparing gypsogenic acid (red) and QA (blue) standards with metabolite extracts from control yeast (carrying empty vectors – pink) and yeast expressing *Saponaria* β‐amyrin synthase (*SoBAS*) together with C28/16α‐oxidase (*SoCYP716A379*) and C23 oxidases (*SoCYP72A984* and *SoCYP72A1003*).
**Figure S12** Alphafold3 models of *S. officinalis* enzymes identified in this study.
**Figure S13** Close up view of transgenic GFP‐expressing hairy roots from *G. elegans* (left) and *S. officinalis* (right) under brightfield (top) or GFP‐enhanced transmission (GFP‐ET) filter (bottom).
**Figure S14** Structures comparison between EEE saponins.
**Figure S15** qPCR results presenting the relative expression of transgenes in different *G. elegans* transgenic hairy root lines overexpressing the control construct (eGFP), each of the four *CYP450*s presented in the study or a combination of the four genes with *SoBAS* (reported as combination).
**Figure S16** Relative quantification of saponins and precursors in transgenic *Gypsophila* hairy root lines expressing *GFP* as control (green), *SoCYP716A378* (blue), *SoCYP716A379* (brown), *SoCYP72A984* (orange), *SoCYP72A1003* (red), or a combination of all CYP450s (purple).
**Figure S17** Vector map of the plasmid used for concomitant expression of *Saponaria* EEE biosynthetic genes identified in this study in *G. elegans* hairy roots.
**Figure S18** EI‐MS fragmentation pattern of trimethylsilyl derivatized triterpenoids β‐amyrin (1), erythrodiol (2), oleanolic acid (3), echinocystic acid (4), caulophyllogenin (5), hederagenin (6), gypsogenin (7), gypsogenic acid and quillaic acid (8), and potential 16‐hydroxy gypsogenic acid (9).

## Data Availability

All data are included in the article, SI, and/or public databanks. RNA‐Seq data have been deposited in the ArrayExpress database (accession E‐MTAB‐14773).
