# Supplementary material for: Engineering Gypsophila elegans hairy root cultures to produce endosomal escape‐enhancing saponins
Source: Plant Biotechnol J. 2025 May 10;23(8):3068–82. doi: 10.1111/pbi.70122 (PMC12310815; doi:10.1111/pbi.70122)
Supplement: Supplementary file 3 — Figure S1. Global transcriptomic changes visualised by multidimensional scaling (MDS), showing gene expression variance between JA‐treated (bottom‐half) and mock‐treated (top half) samples, as well as across tissues (leaves, stems, roots). Figure S2 Count per millions (CPM) expression values in Saponaria RNA‐seq data for all candidate gene transcripts considered in this study across different tissues. Figure S3 Heatmap reporting scaled expression values (mean‐centered) from Saponaria RNA‐seq data of the SOM cluster 65 (i.e. node) containing SoBAS and SoCYP450s characterised in this study (in bold). Figure S4 Summary of the NMR analysis of the samples containing 16α‐hydroxygypsogenic acid. Figure S5 1H NMR spectrum of the sample containing 16α‐hydroxygypsogenic acid. Figure S6 1H‐13C HSQC spectrum of the sample containing 16α‐hydroxy‐gypsogenic acid. Figure S7 HMBC spectrum of the sample containing 16α‐hydroxy‐gypsogenic acid. Figure S8 Flow cytrometry of DAPI‐stained nuclei from Arabidopsis, maize and S. officinalis. Figure S9 ClustalW comparison of protein sequences of the Saponaria CYP450s identified in this study. Figure S10 Heatmap reporting scaled expression values (mean‐centered) from Saponaria RNA‐seq data of the SOM cluster 144 (i.e. node) containing SoCYP72A1003 (in bold). Rows represent gene transcripts and columns represent experimental tissues/treatments. Figure S11 LC‐MS XIC chromatograms comparing gypsogenic acid (red) and QA (blue) standards with metabolite extracts from control yeast (carrying empty vectors – pink) and yeast expressing Saponaria β‐amyrin synthase (SoBAS) together with C28/16α‐oxidase (SoCYP716A379) and C23 oxidases (SoCYP72A984 and SoCYP72A1003). Figure S12 Alphafold3 models of S. officinalis enzymes identified in this study. Figure S13 Close up view of transgenic GFP‐expressing hairy roots from G. elegans (left) and S. officinalis (right) under brightfield (top) or GFP‐enhanced transmission (GFP‐ET) filter (bottom). Figure S14 Struc [file PBI-23-3068-s003.docx]

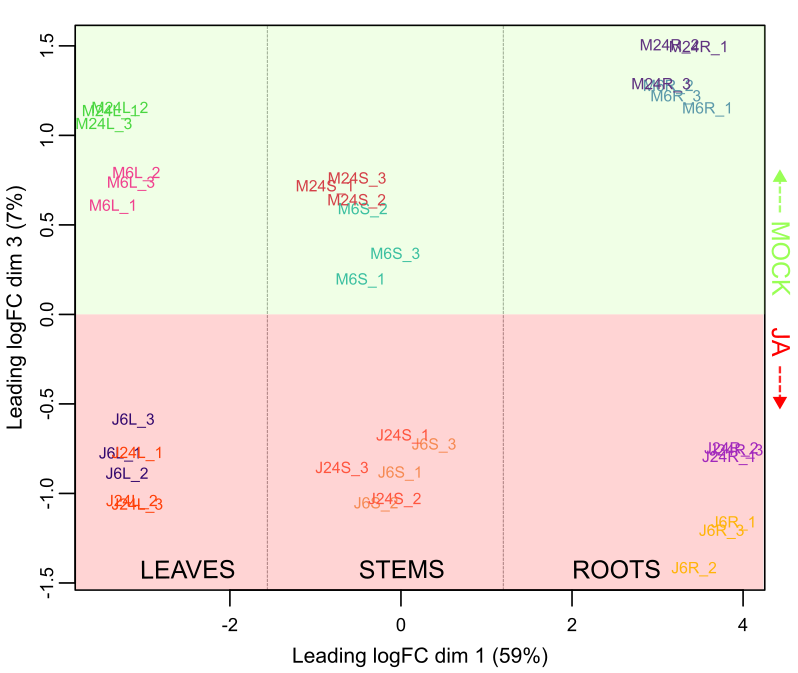


**Figure S1:** Global transcriptomic changes visualized by multidimensional scaling (MDS), showing gene expression variance between JA-treated (bottom-half) and mock-treated (top half) samples, as well as across tissues (leaves, stems, roots). Distinct clusters indicate differential expression profiles linked to treatment and tissue type. Codes represent different datasets: J = JA-treated, M = mock, 6/24 = h after treatment, L = leaves, S = stems, R = roots, 1/2/3 = biological replica.

**
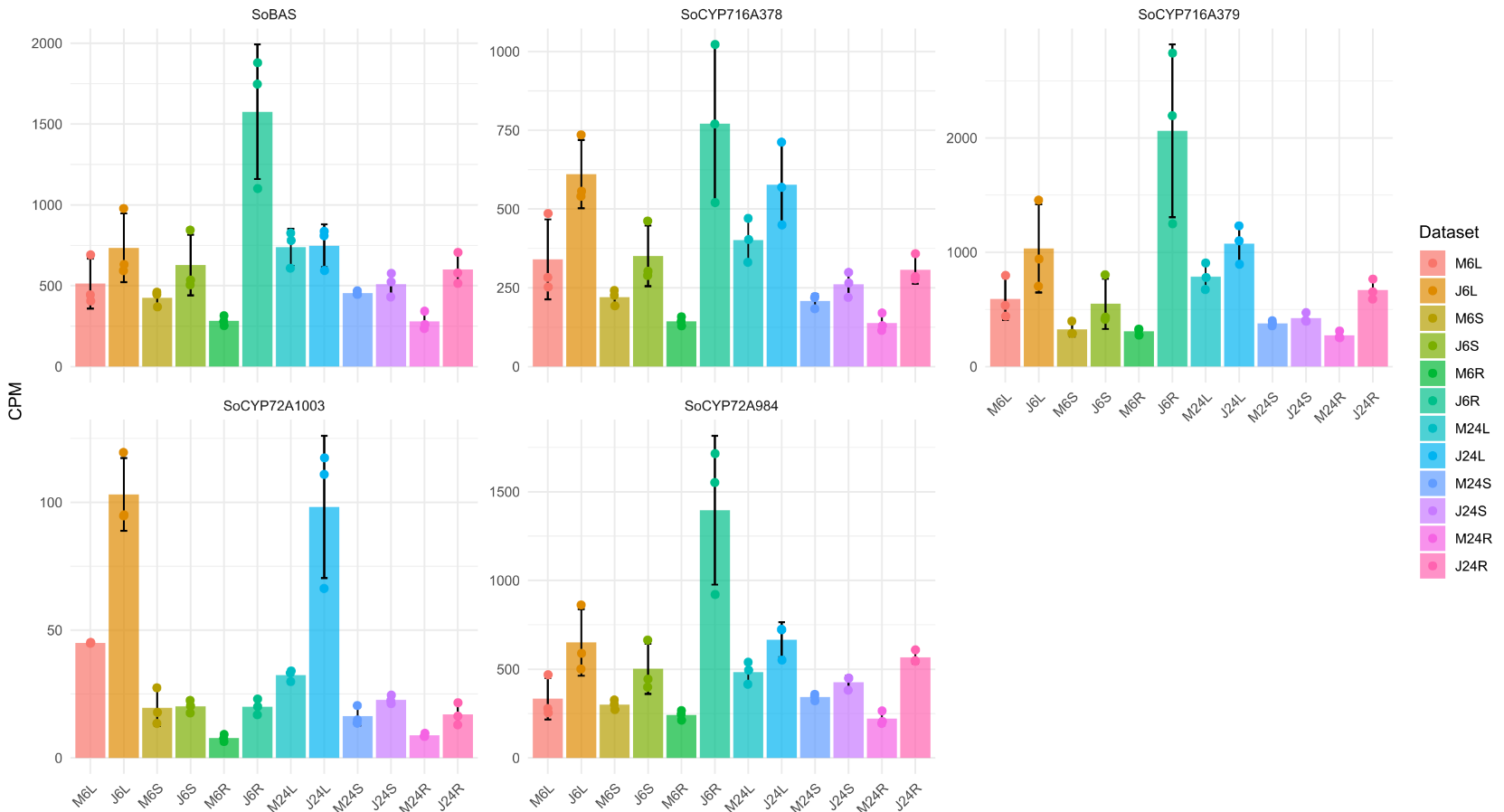
**

**Figure S2:** Count per millions (CPM) expression values in *Saponaria* RNA-seq data for all candidate gene transcripts considered in this study across different tissues. J = JA-treated, M = mock, 6/24 = h after treatment, L = leaves, S = stems, R = roots.


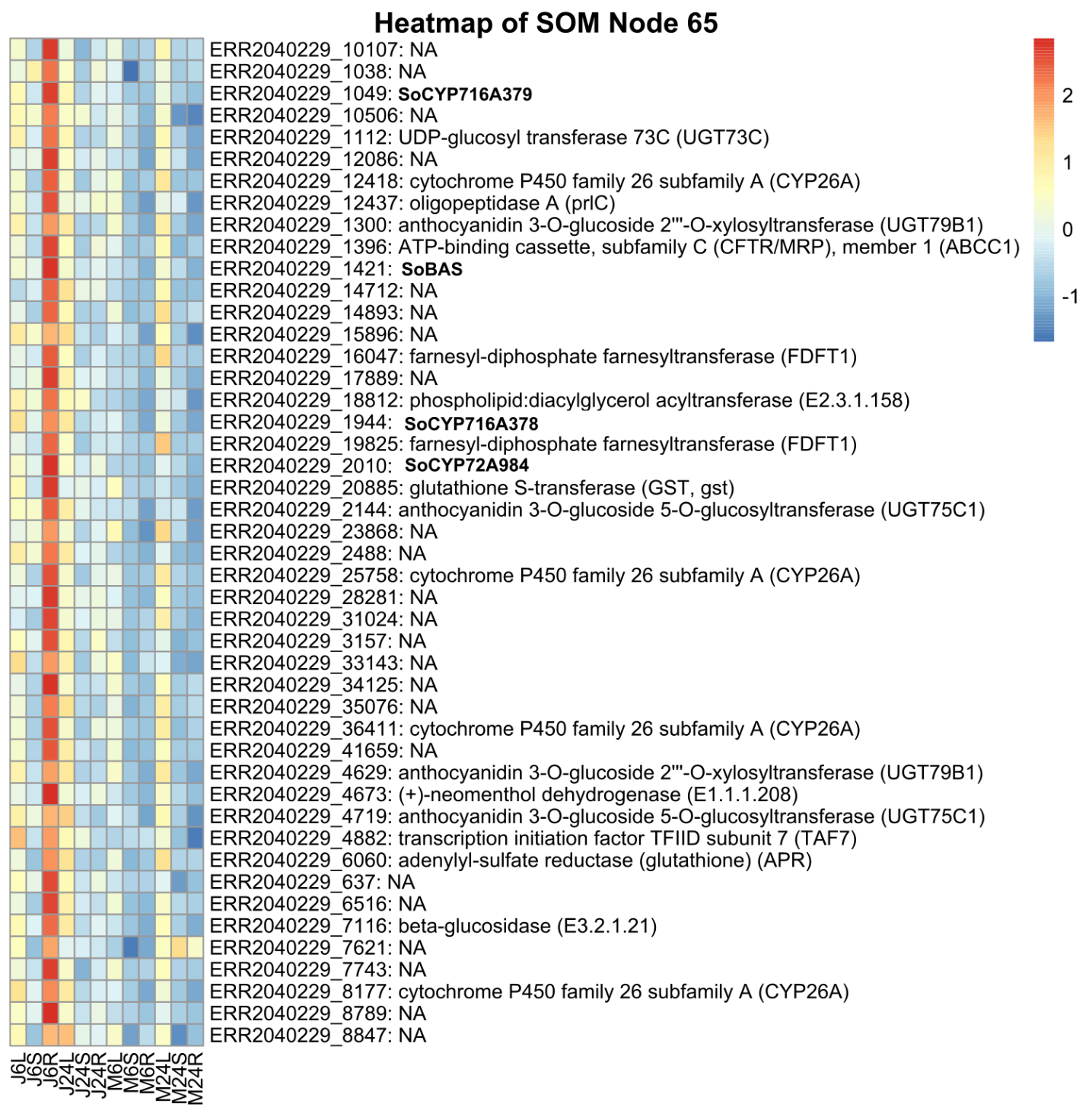


**Figure S3:** Heatmap reporting scaled expression values (mean-centered) from *Saponaria* RNA-seq data of the SOM cluster 65 (i.e. node) containing *SoBAS* and *SoCYP450s* characterized in this study (in bold). Rows represent gene transcripts and columns represent experimental tissues/treatments. J = JA-treated, M = mock, 6/24 = h after treatment, L = leaves, S = stems, R = roots. NA = transcripts for which description by TRAPID (Van Bel et al., 2013) was not available.


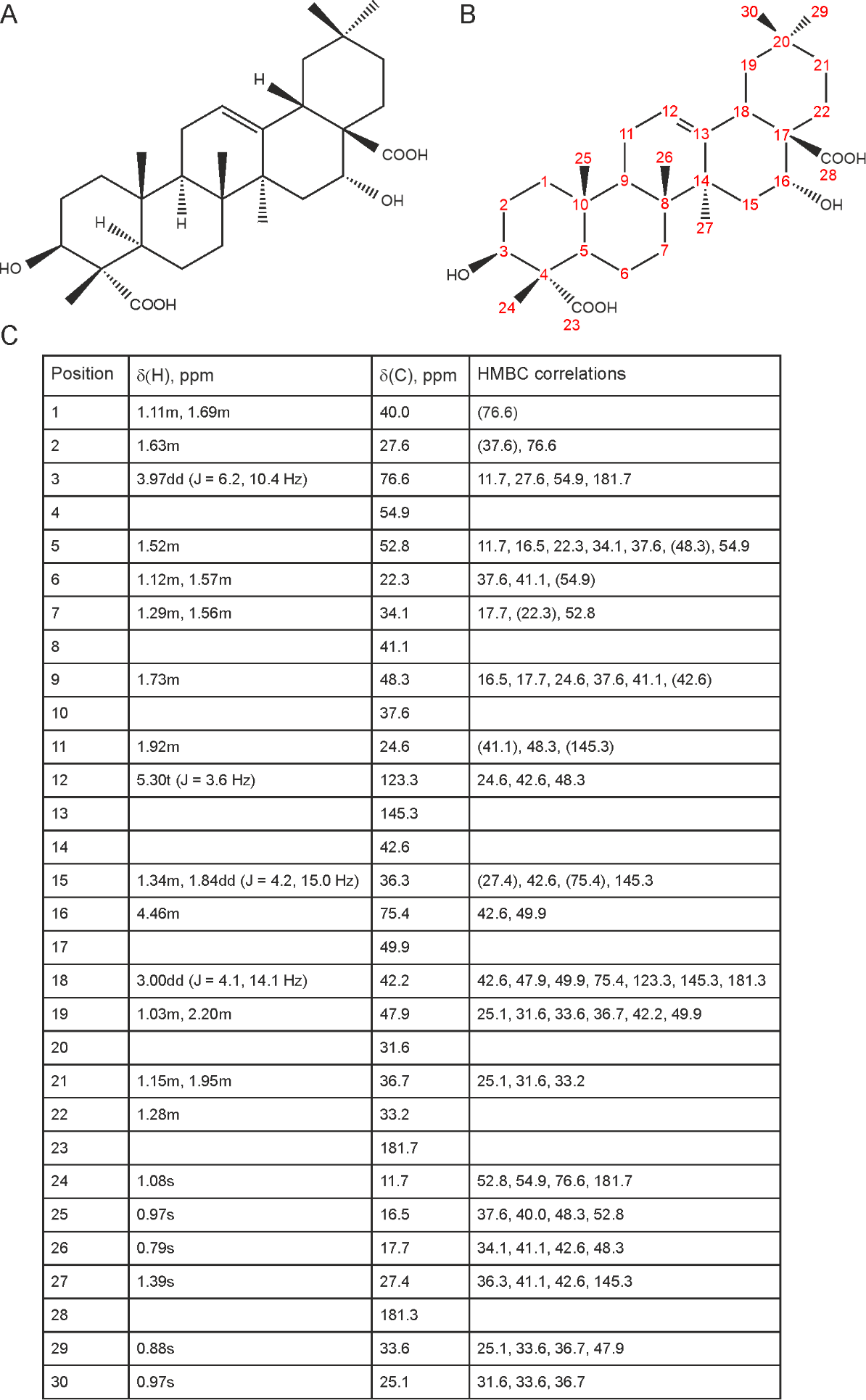


**Figure S4.** Summary of the NMR analysis of the samples containing 16α-hydroxygypsogenic acid. A) Structural formula, B) atom numbering scheme, and C) NMR assignments of 16α-hydroxygypsogenic acid. Multiplicities are denoted as: s – singlet, dd – doublet of doublets, t – triplet, m – multiplet. Weak HMBC correlations are given between parentheses. **
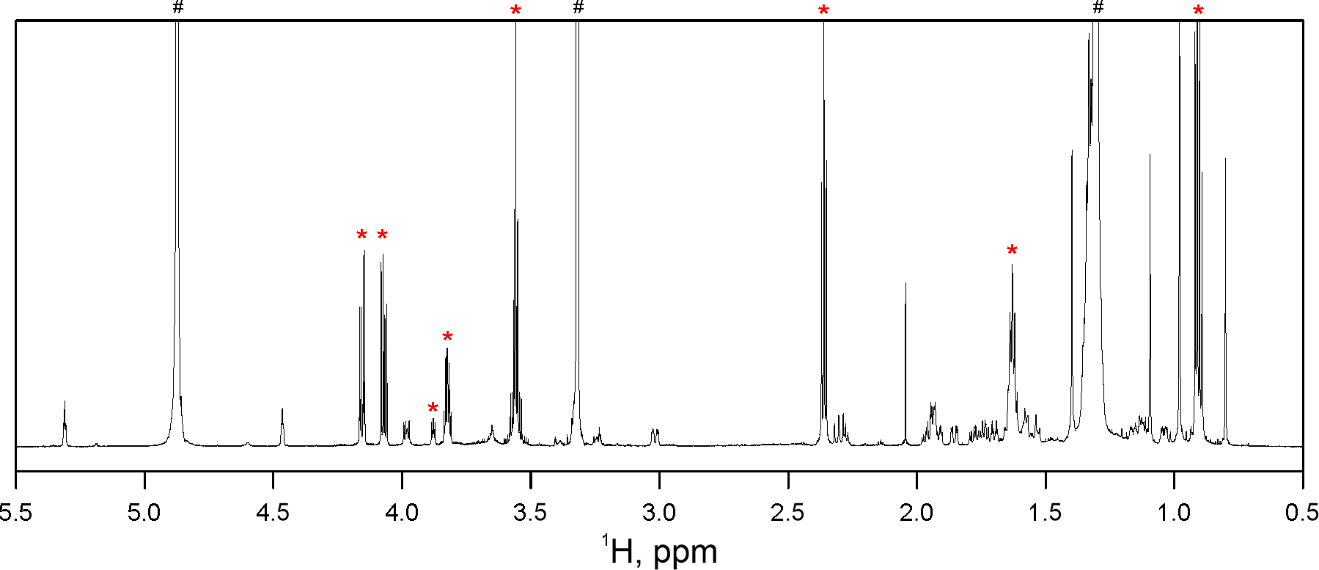
**

**Figure S5.** ^1^H NMR spectrum of the sample containing 16α-hydroxygypsogenic acid. The asterisks identify signals of a dominant contaminant observed in all measured samples, while the hash symbols indicate residual solvent peaks at 4.87 ppm (H_2_O) and 3.31 ppm (CH_3_OH) and an impurity at 1.29 ppm.


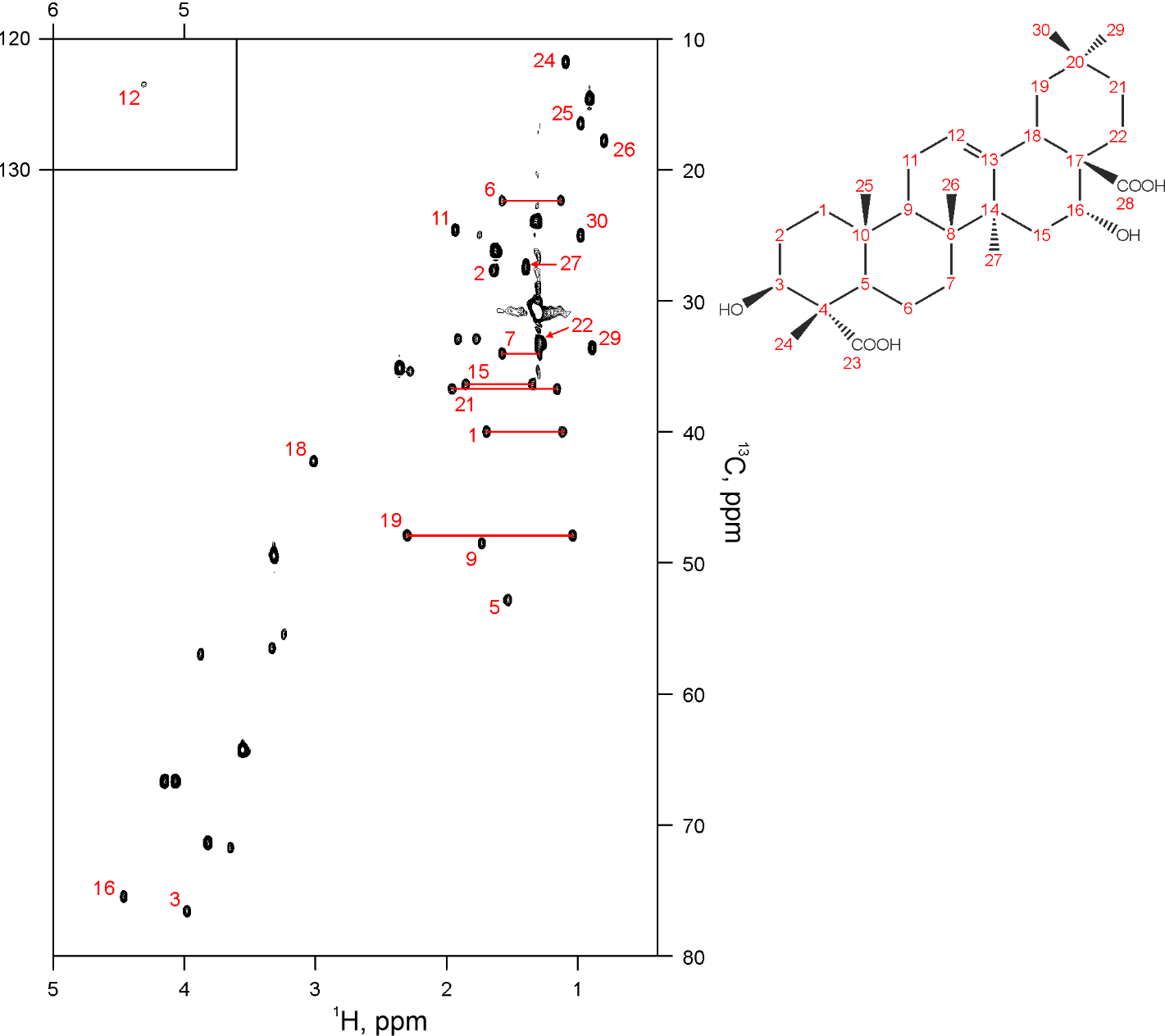


**Figure S6.** ^1^H-^13^C HSQC spectrum of the sample containing 16α-hydroxy-gypsogenic acid. The resonances of 16α-hydroxy-gypsogenic acid are labelled according to the atom numbering scheme. Horizontal lines identify pairs of peaks arising from hydrogens bound to the same carbon atom (CH_2_ groups). The inset shows a downfield shifted alkene resonance.


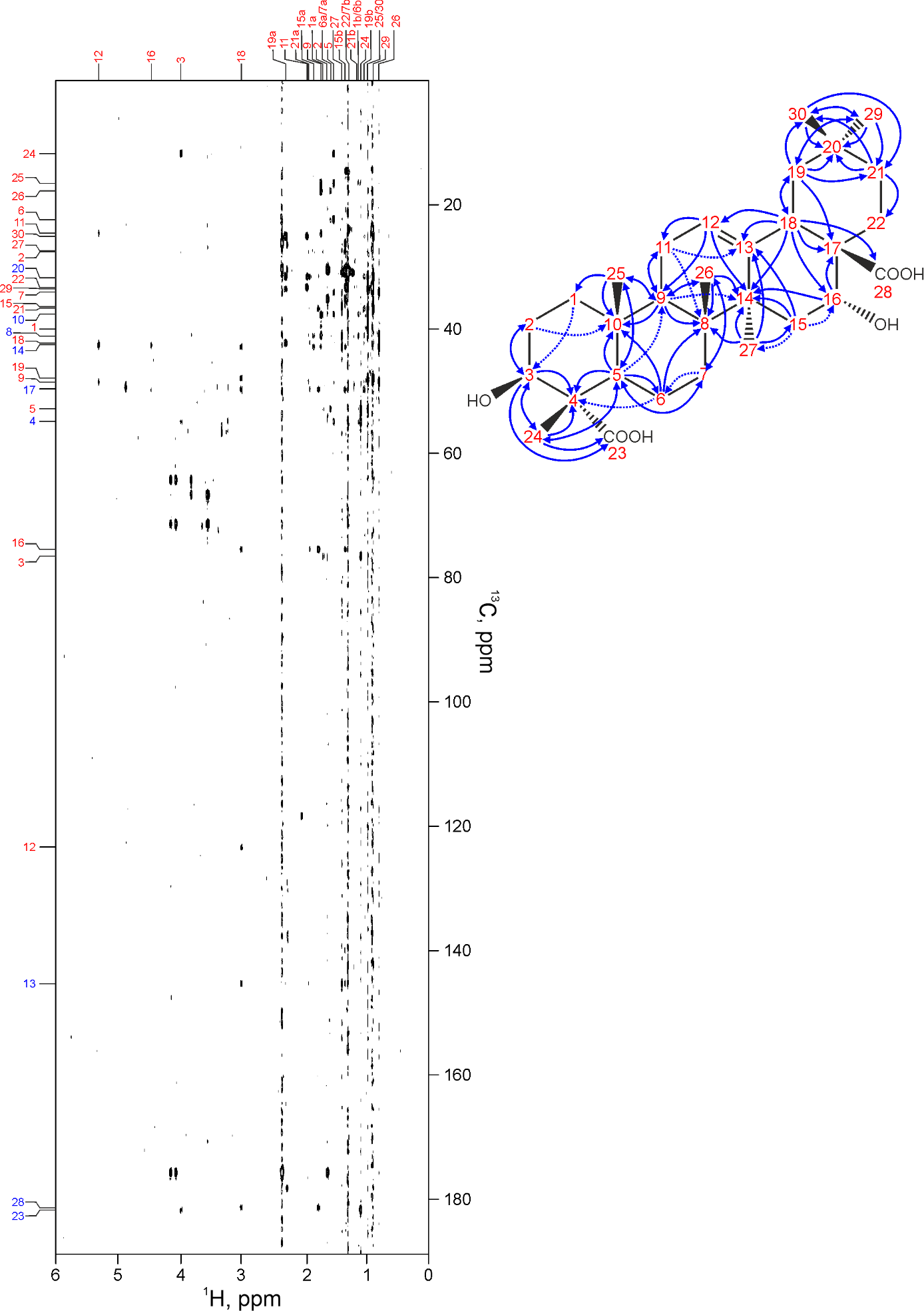


**Figure S7.** HMBC spectrum of the sample containing 16α-hydroxy-gypsogenic acid. Positions of proton and carbon resonances of 16α-hydroxygypsogenic acid are indicated in the plot, with quaternary carbons shown in blue. The observed H→C correlations are illustrated in the structure (right), with dashed lines denoting possible connections arising from weak signals (corresponding to the values in parentheses in Figure S1C).


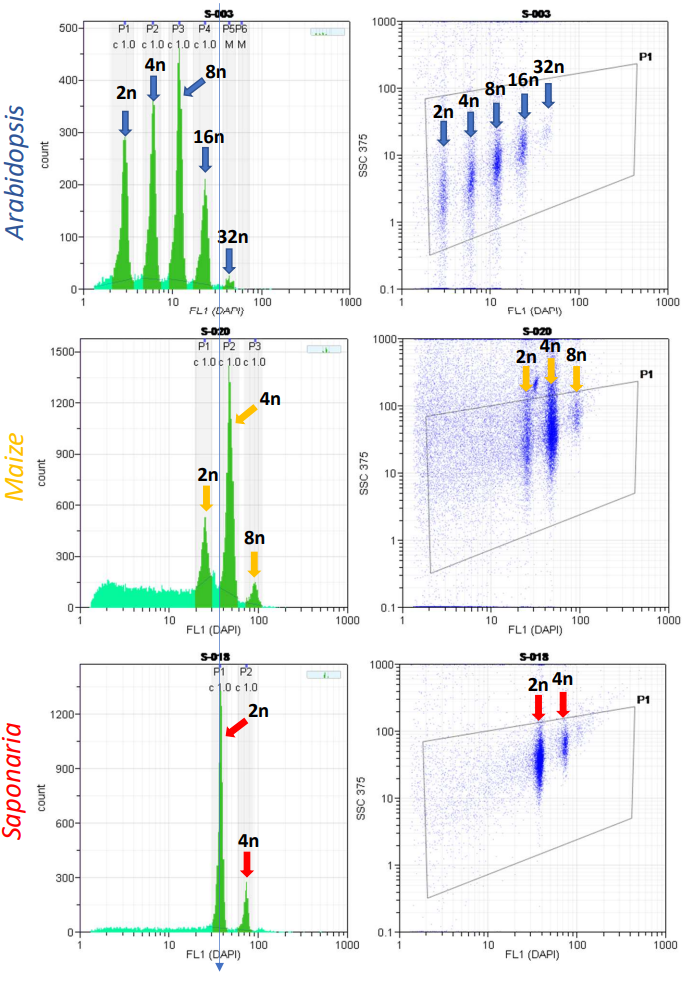


**Figure S8:** Flow cytrometry of DAPI-stained nuclei from *Arabidopsis*, maize and *S. officinalis*. The comparison allowed to estimate the genome size of *Saponaria* by comparing the fluorescent signal to those of maize and *Arabidopsis*. Arrows point to main peaks associated with diploid or polyploid cells.


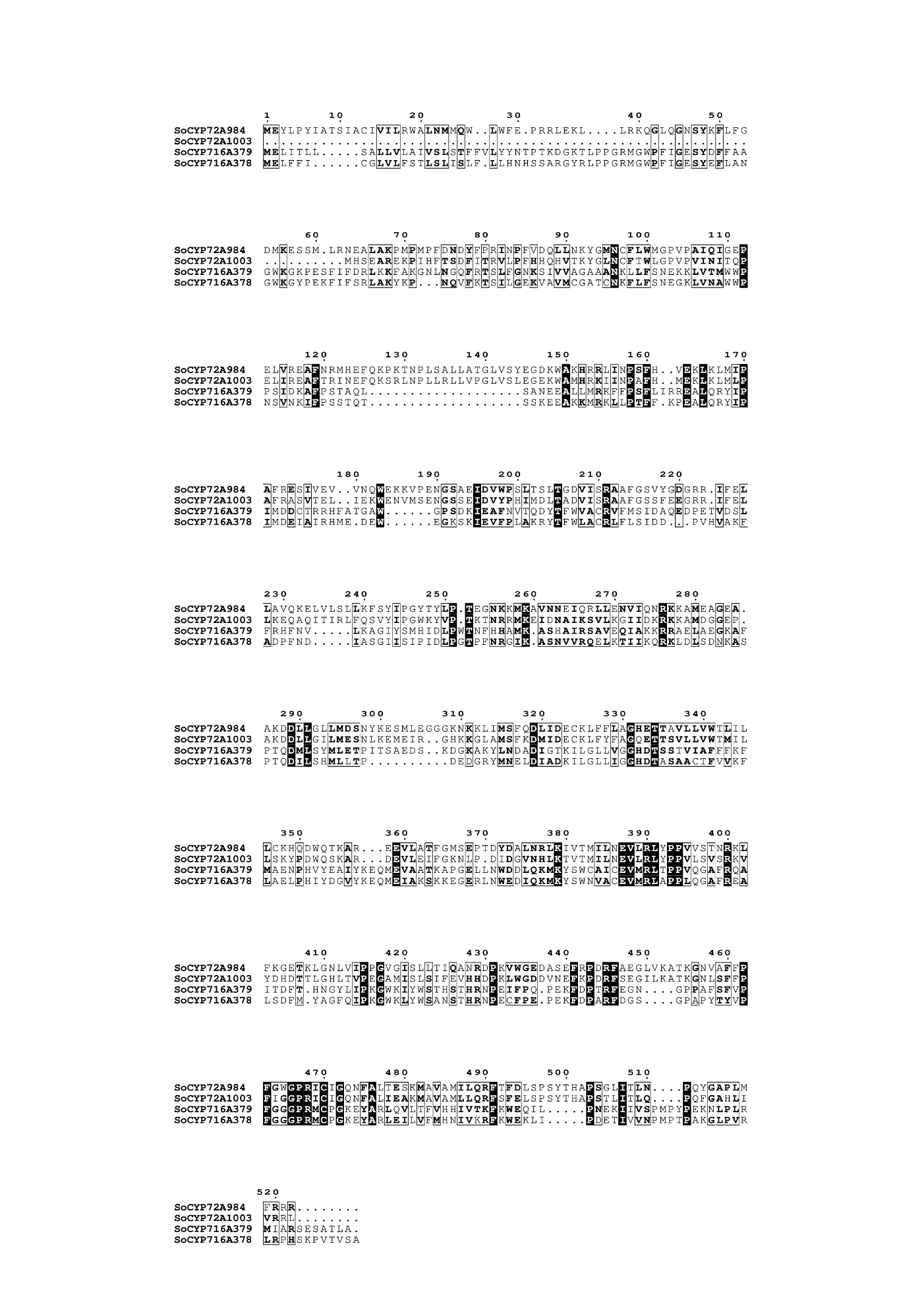


**Figure S9**: ClustalW comparison of protein sequences of the *Saponaria* CYP450s identified in this study.


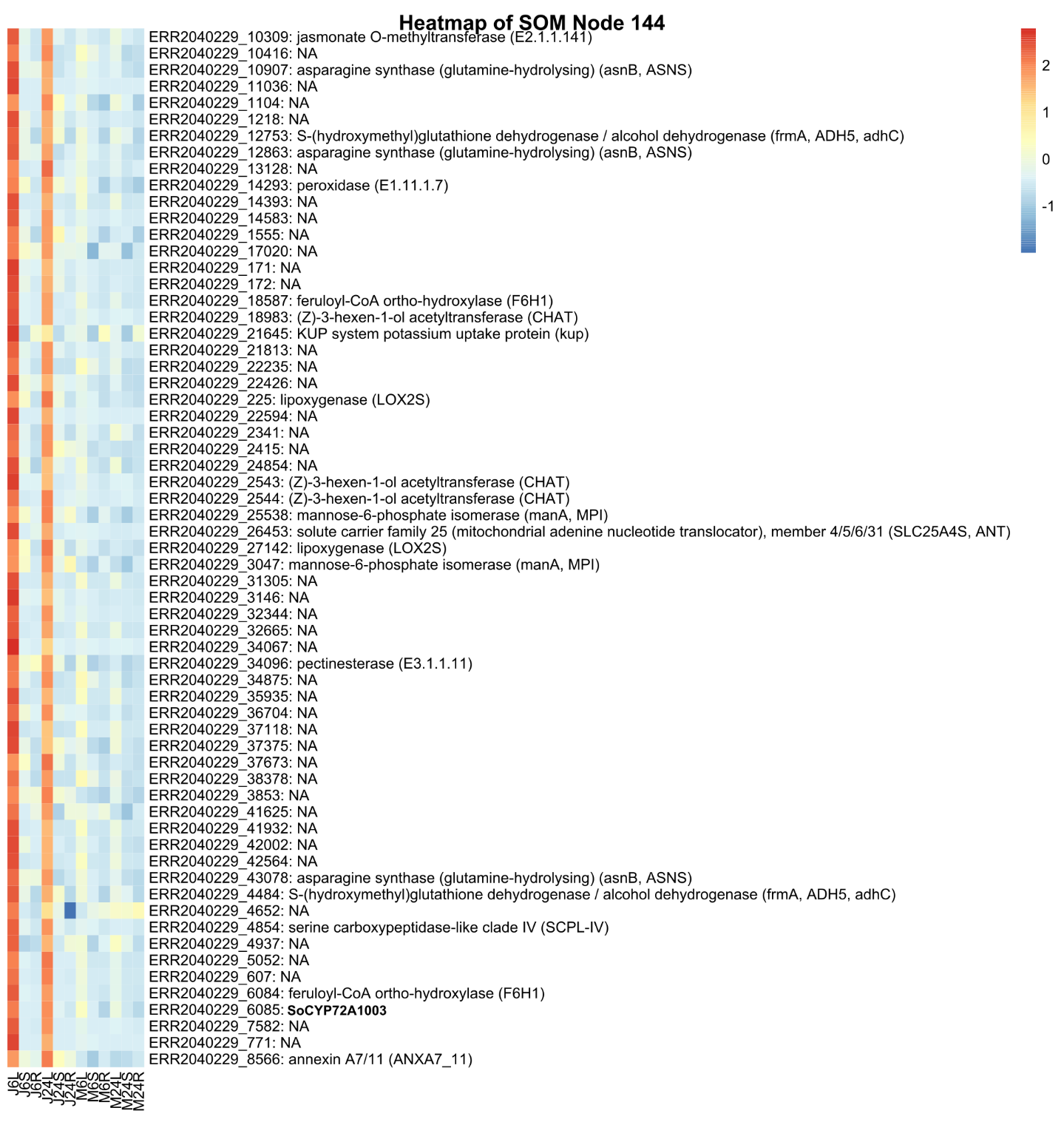


**Figure S10:** Heatmap reporting scaled expression values (mean-centered) from *Saponaria* RNA-seq data of the SOM cluster 144 (i.e. node) containing *SoCYP72A1003* (in bold). Rows represent gene transcripts and columns represent experimental tissues/treatments. J = JA-treated, M = mock, 6/24 = h after treatment, L = leaves, S = stems, R = roots. NA= transcripts for which description by TRAPID (Van Bel et al., 2013) was not available.


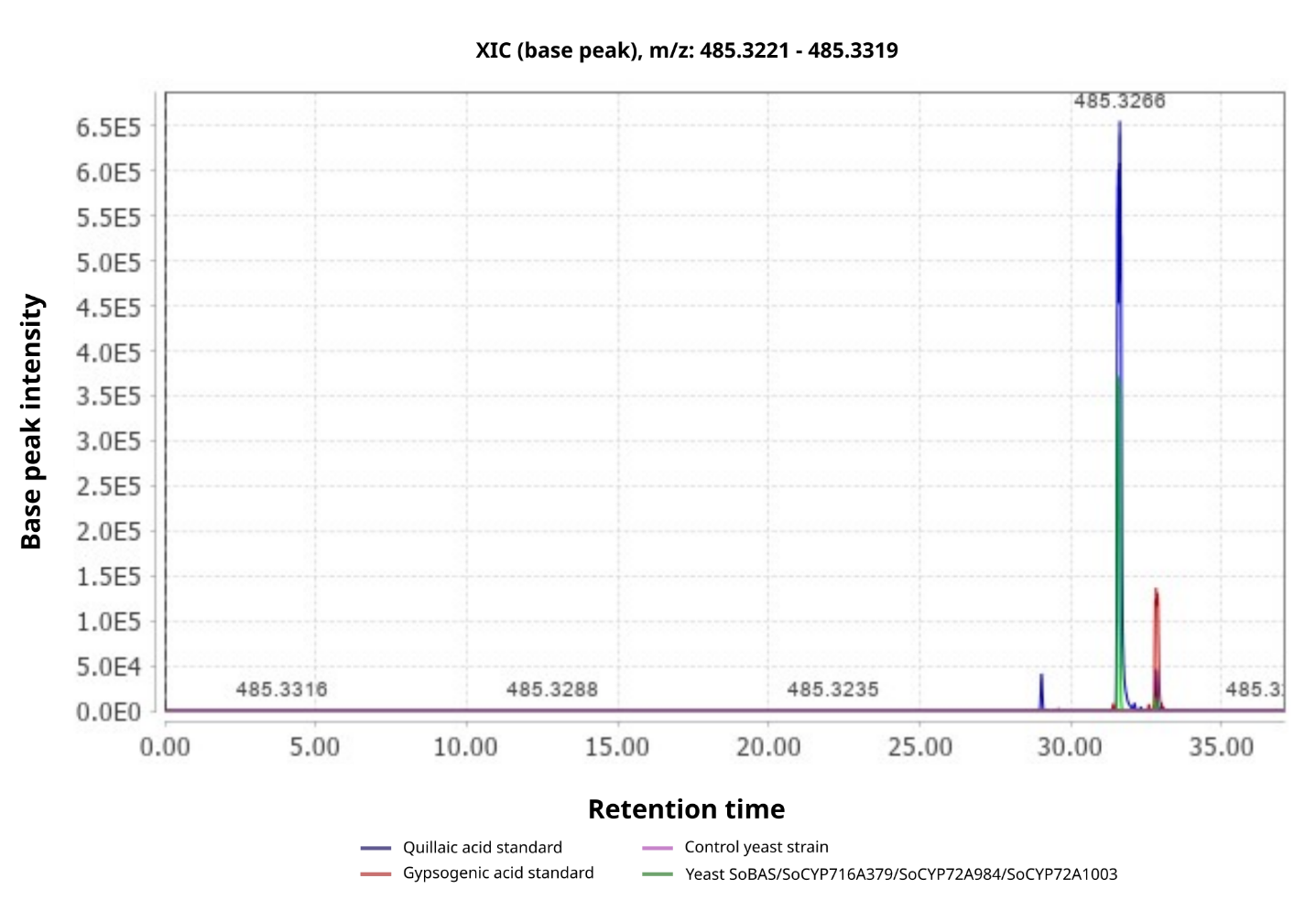


**Figure S11:** LC-MS XIC chromatograms comparing gypsogenic acid (red) and QA (blue) standards with metabolite extracts from control yeast (carrying empty vectors – pink) and yeast expressing *Saponaria* β-amyrin synthase (*SoBAS*) together with C28/16α-oxidase (*SoCYP716A379*) and C23 oxidases (*SoCYP72A984* and *SoCYP72A1003*). The overlap between the blue and green peaks demonstrates QA production in the transgenic yeast expressing *Saponaria* enzymes.


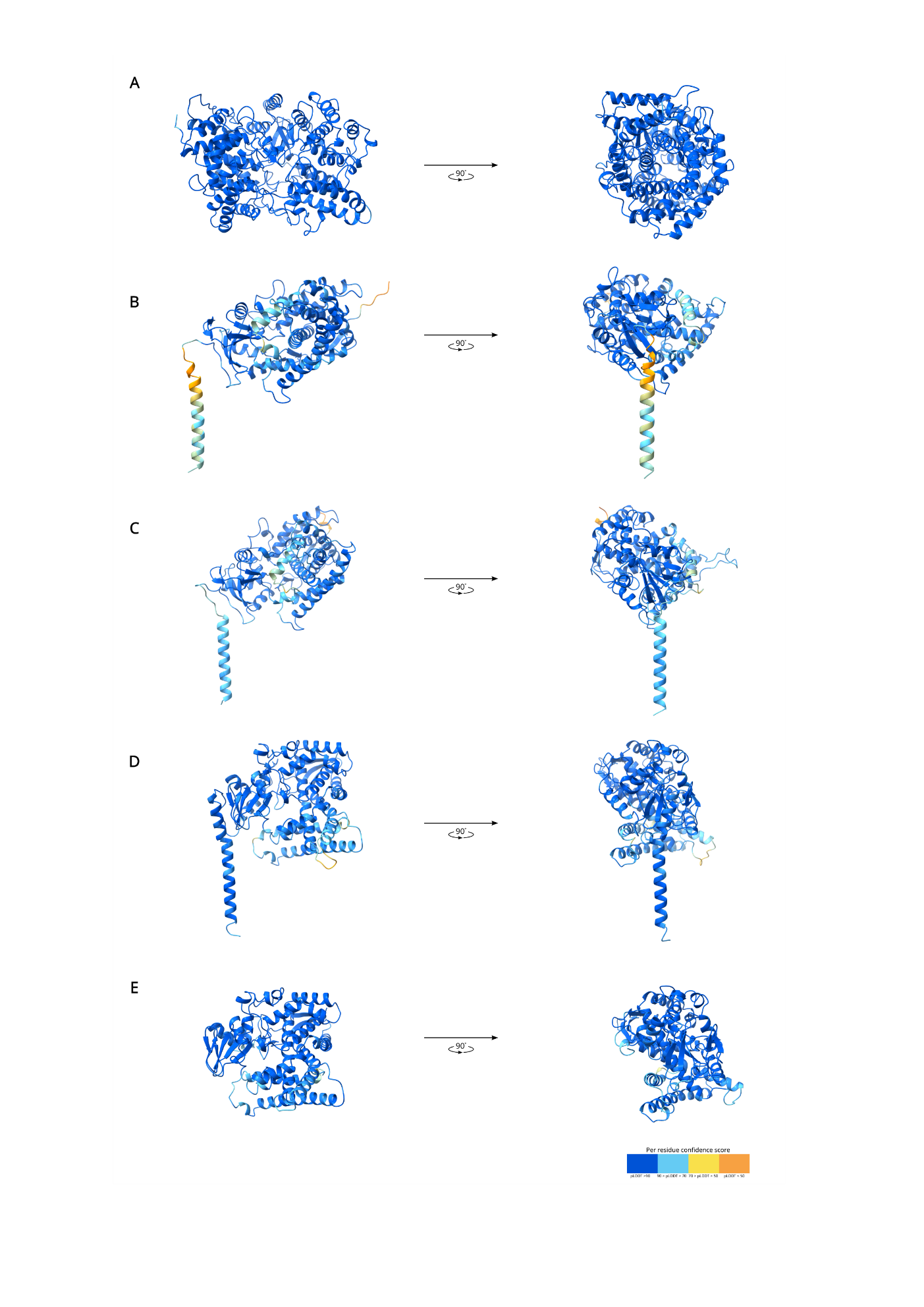


**Figure S12:** Alphafold3 models of *S. officinalis* enzymes identified in this study. A) SoBAS, B) SoCYP716A378 C-28 oxidase, C) SoCYP716A379 C-16α/28 oxidase, D) SoCYP72A984 C-23 oxidase, E) SoCYP72A1003 C-23 oxidase. Models are presented in two views reciprocally turned by 90 degrees. In the bottom right corner, the key color legend related to the per-residue pLDDT confidence score is indicated.


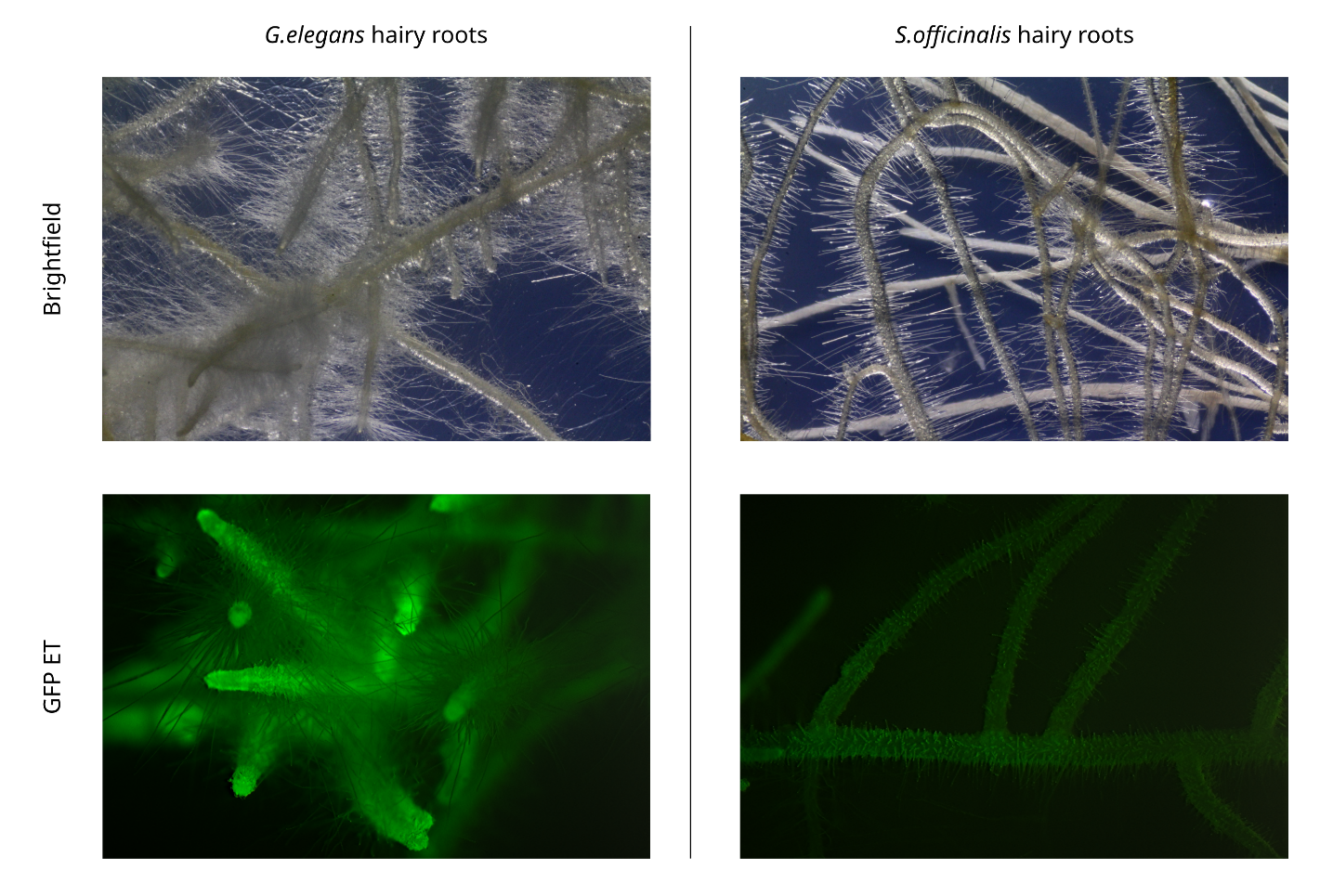


**Figure S13:** Close up view of transgenic GFP-expressing hairy roots from *G. elegans* (left) and *S. officinalis* (right) under brightfield (top) or GFP-enhanced transmission (GFP-ET) filter (bottom).


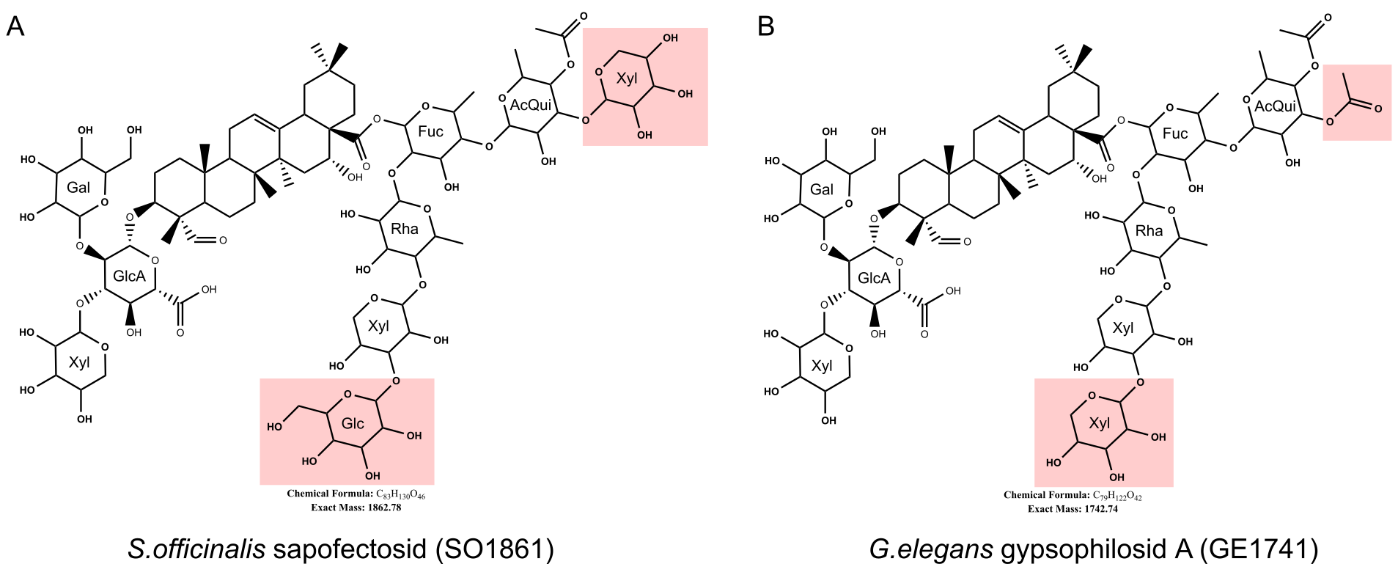


**Figure S14:** Structures comparison between EEE saponins. A) sapofectosid from *S. officinalis* (i.e. SO1861) and B) gypsophilosid from *G. elegans* (i.e. GE1741) (Sama et al., 2018; Sama et al., 2017). Substituents differing between the two molecules are highlighted in light-red. The corresponding brute formula and calculated exact mass are reported below each structure.

**
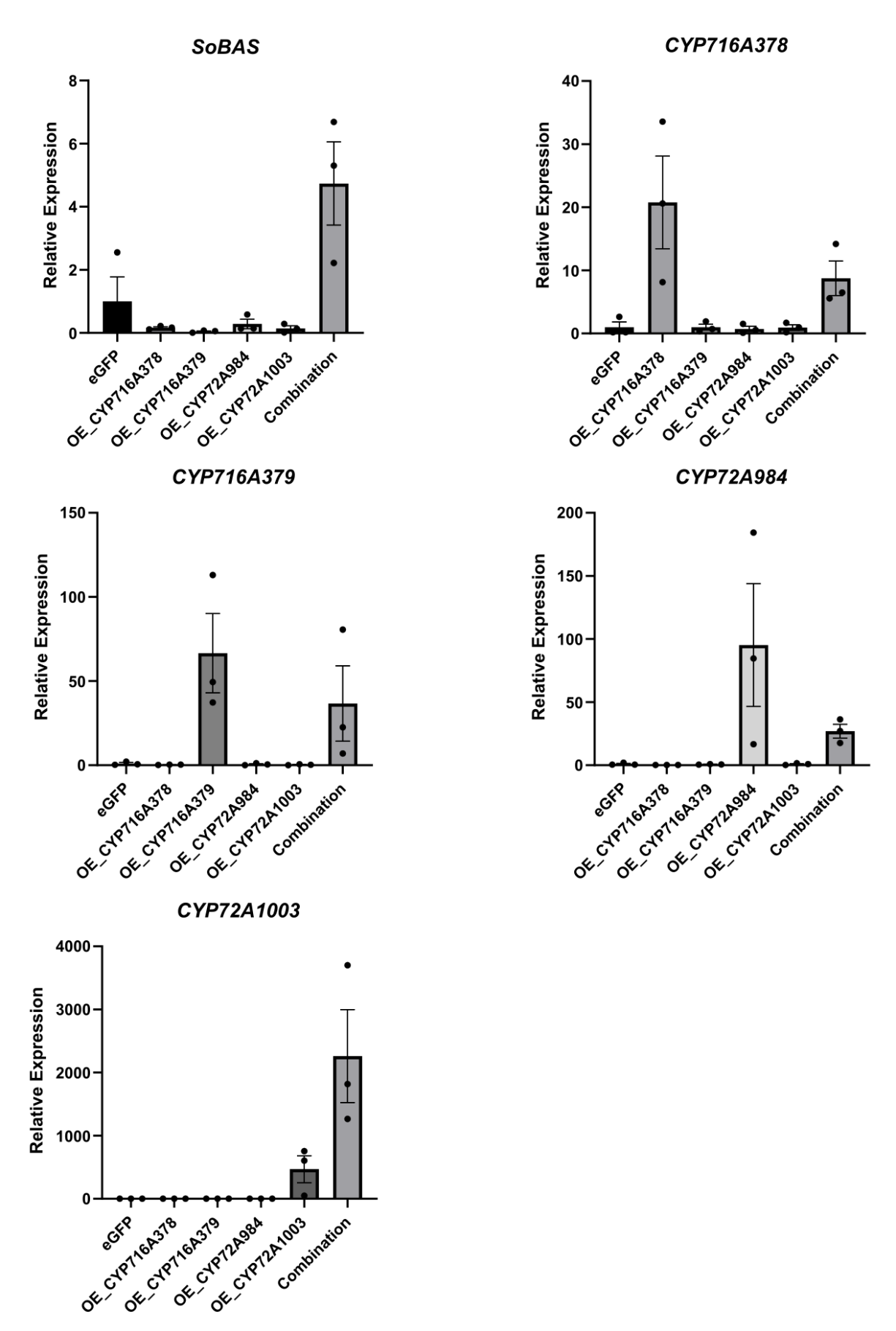
**

**Figure S15:** qPCR results presenting the relative expression of transgenes in different *G. elegans* transgenic hairy root lines overexpressing the control construct (eGFP), each of the four *CYP450*s presented in the study or a combination of the four genes with *SoBAS* (reported as combination).


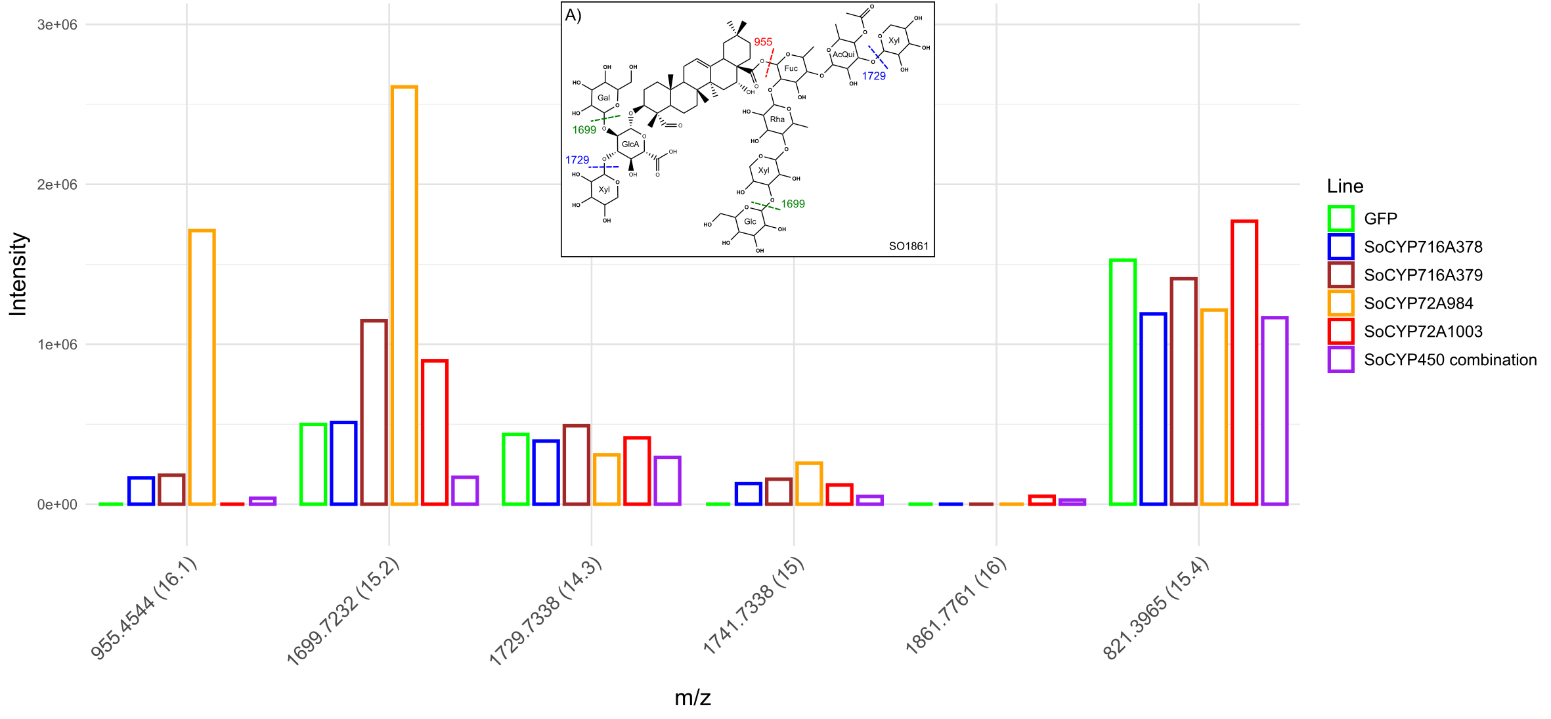


**Figure S16:** Relative quantification of saponins and precursors in transgenic *Gypsophila* hairy root lines expressing *GFP* as control (green), *SoCYP716A378* (blue), *SoCYP716A379* (brown), *SoCYP72A984* (orange), *SoCYP72A1003* (red), or a combination of all CYP450s (purple). Accurate mass (*m/z*) are reported on the x-axis and, within between parentheses, their respective retention time in min. The reported m/z values correspond to gypsophilosid (*m/z* 1741.7338), the native saponin produced by *Gypsophila* (structure reported in Figure S14), sapofectosid (SO1861) and its biosynthetic precursors without terminal xylose (*m/z* 1729.7338), without terminal hexose (*m/z* 1699.7232), and without C28 glycosides (*m/z* 955.4544), as schematized in panel A). The *m/z* 821.3965 corresponds to glycyrrhizin that was used as an internal standard to control for extraction efficiency during saponin extraction from the hairy root lines.


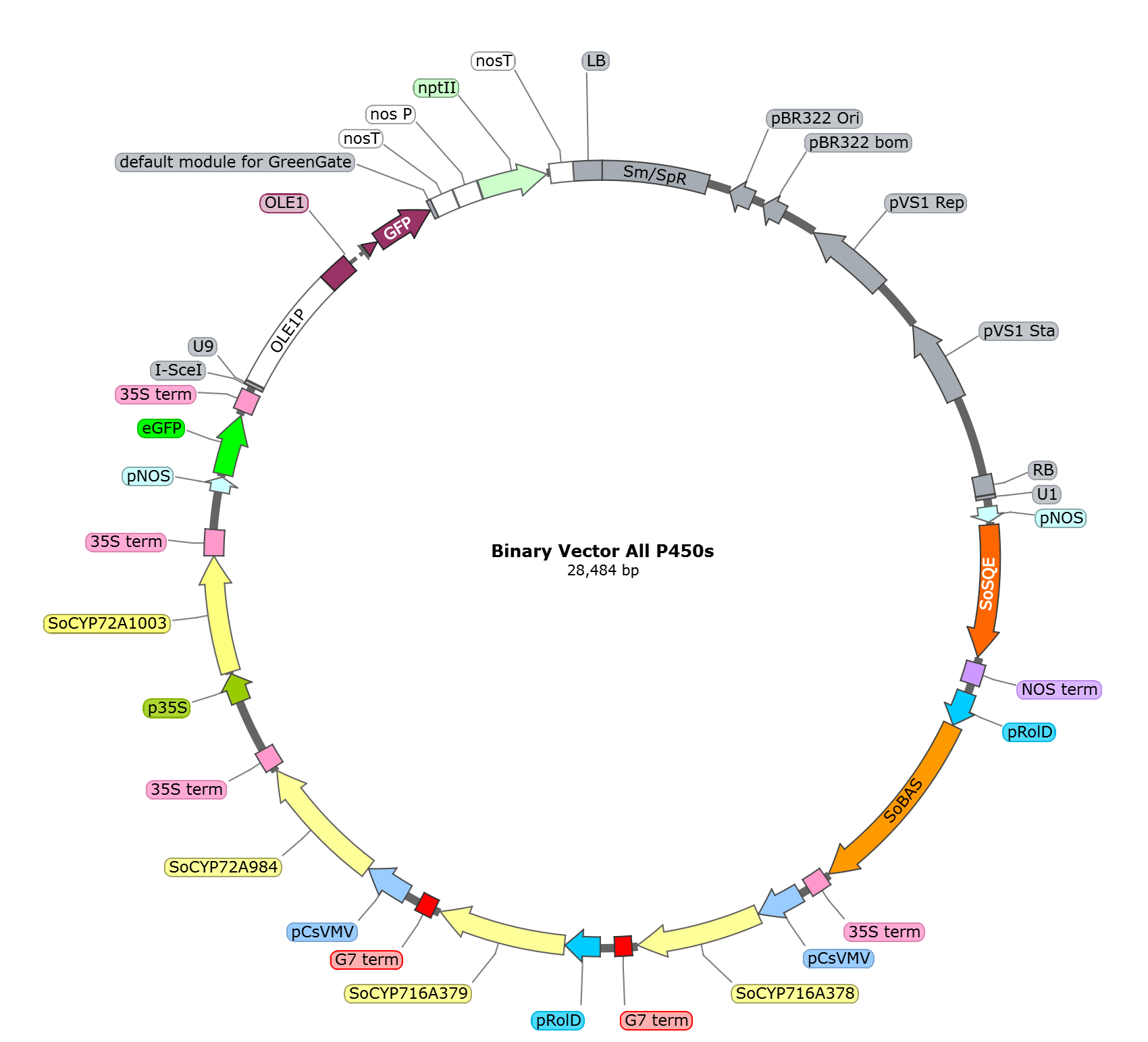


**Figure S17:** Vector map of the plasmid used for concomitant expression of *Saponaria* EEE biosynthetic genes identified in this study in *G. elegans* hairy roots. Backbone and modules are available at <https://vectorvault.vib.be/>


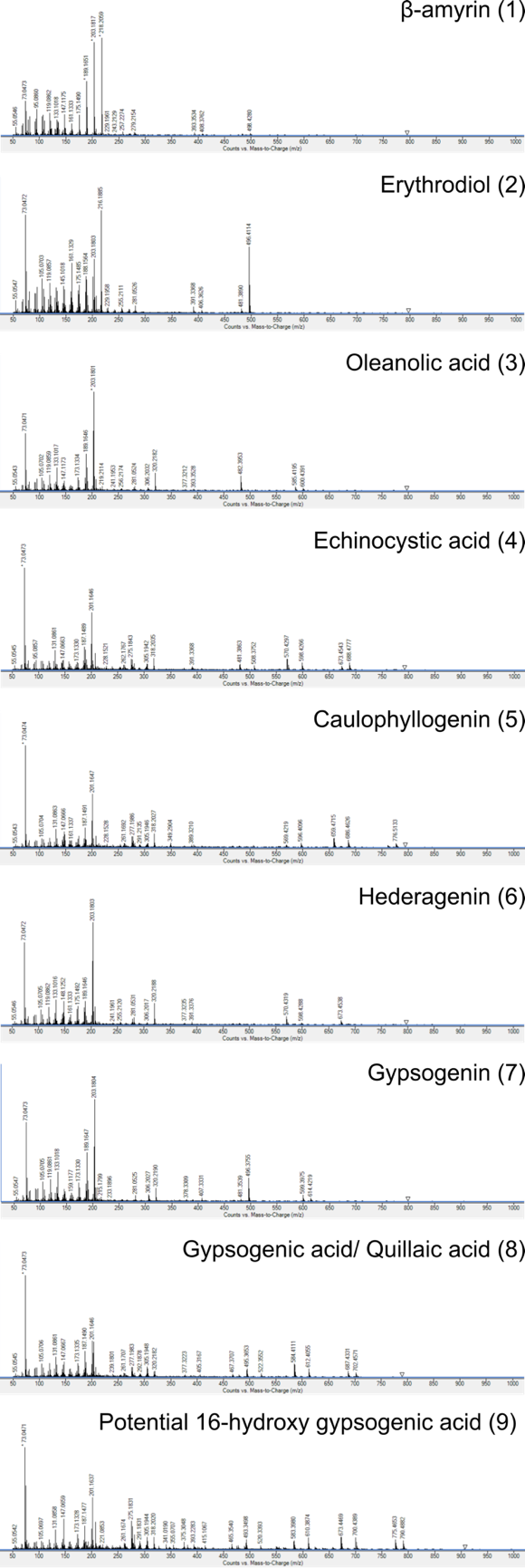


**Figure S18:** EI-MS fragmentation pattern of trimethylsilyl derivatized triterpenoids β-amyrin (1), erythrodiol (2), oleanolic acid (3), echinocystic acid (4), caulophyllogenin (5), hederagenin (6), gypsogenin (7), gypsogenic acid and quillaic acid (8), and potential 16-hydroxy gypsogenic acid (9).
